# Discovery of potent necroptosis inhibitors targeting RIPK1 kinase activity for the treatment of inflammatory disorder and cancer metastasis

**DOI:** 10.1038/s41419-019-1735-6

**Published:** 2019-06-24

**Authors:** Jue Hou, Jie Ju, Zili Zhang, Cong Zhao, Zhanhui Li, Jiyue Zheng, Tian Sheng, Hongjian Zhang, Linkun Hu, Xiaoliang Yu, Wei Zhang, Yangxin Li, Meng Wu, Haikuo Ma, Xiaohu Zhang, Sudan He

**Affiliations:** 1 Cyrus Tang Hematology Center and Collaborative Innovation Center of Hematology, State Key Laboratory of Radiation Medicine and Protection, Soochow University, 215123 Suzhou, Jiangsu China; 2 Key Laboratory of Stem Cells and Biomedical Materials of Jiangsu Province and Chinese Ministry of Science and Technology, Soochow University, 215123 Suzhou, Jiangsu China; 3 Jiangsu Key Laboratory of Neuropsychiatric Diseases and College of Pharmaceutical Sciences, Soochow University, 215123 Suzhou, Jiangsu China; 4 Department of Urology, The First Affiliated Hospital of Soochow University, 188 Shizi Rd, 215006 Suzhou, Jiangsu China; 5 Center of Systems Medicine, Institute of Basic Medical Sciences, Chinese Academy of Medical Sciences & Peking Union Medial College, Beijing, 100005 China; 6 Suzhou Institute of Systems Medicine, 215123 Suzhou, Jiangsu China; 7 Institute for Cardiovascular Science and Department of Cardiovascular Surgery, The First Affiliated Hospital of Soochow University, Suzhou, Jiangsu China

**Keywords:** Necroptosis, Target validation

## Abstract

Necroptosis is a form of regulated necrosis controlled by receptor-interacting kinase 1 (RIPK1 or RIP1), RIPK3 (RIP3), and pseudokinase mixed lineage kinase domain-like protein (MLKL). Increasing evidence suggests that necroptosis is closely associated with pathologies including inflammatory diseases, neurodegenerative diseases, and cancer metastasis. Herein, we discovered the small-molecule PK6 and its derivatives as a novel class of necroptosis inhibitors that directly block the kinase activity of RIPK1. Optimization of PK6 led to PK68, which has improved efficacy for the inhibition of RIPK1-dependent necroptosis, with an EC_50_ of around 14–22 nM in human and mouse cells. PK68 efficiently blocks cellular activation of RIPK1, RIPK3, and MLKL upon necroptosis stimuli. PK68 displays reasonable selectivity for inhibition of RIPK1 kinase activity and favorable pharmacokinetic properties. Importantly, PK68 provides strong protection against TNF-α-induced systemic inflammatory response syndrome in vivo. Moreover, pre-treatment of PK68 significantly represses metastasis of both melanoma cells and lung carcinoma cells in mice. Together, our study demonstrates that PK68 is a potent and selective inhibitor of RIPK1 and also highlights its great potential for use in the treatment of inflammatory disorders and cancer metastasis.

## Introduction

Necroptosis is a form of regulated necrosis that is tightly controlled by the activation of receptor-interacting protein kinases (RIPKs)^1^. In response to initiating necroptotic signals from death receptors, Toll-like receptor or interferon receptors, RIPK1 (RIP1) and RIPK3 (RIP3), form a protein complex through their RIP homotypic interaction motif (RHIM) domains, leading to activation of both proteins necroptosis^2–5^. The kinase activities of RIPK1 and RIPK3 are essential for their function in necroptosis. Activated RIPK3 phosphorylates its substrate MLKL^6,7^, which further oligomerizes and translocates to the plasma membrane for necroptosis execution^8–10^. Necroptosis results in cell membrane rupture and the release of cellular contents including damage-associated molecular molecules (DAMPs), thus leading to the induction of inflammation. Necroptosis is emerging as an important form of cell death involved in a wide variety of inflammatory or degenerative disorders including tumor necrosis factor (TNF)-induced systemic inflammatory response syndrome (SIRS)^11,12^, systemic inflammatory responses in A20^−/−^ mice^13,14^, chronic proliferative dermatitis (cpdm) in SHARPIN^−/−^ mice^12^, colitis^15^, neurodegenerative diseases^16^ and ischemia–reperfusion-induced injury in the brain^17^, kidney^18,19^, and heart^20,21^^.^ In addition, the necroptosis of endothelial cells has been reported to promote tumor metastasis by facilitating extravasation of circulating tumor cells from the blood system^22^. Therefore, interventions targeting the necroptosis signaling pathway could potentially be used for the treatment the aforementioned necroptosis-associated diseases.

RIPK1 is a multifunctional protein involved in the regulation of cell death and pro-survival nuclear factor-κB (NF-κB) signaling. RIPK1 contains an N-terminal serine/threonine kinase domain, a C-terminal death domain (DD), and an intermediate domain (ID) that contains an RHIM domain. The kinase activity of RIPK1 is essential for its role in mediating necroptosis, whereas this activity is dispensable for RIPK1’s function in NF-κB activation^5,23^. Extensive studies have shown that inhibition of RIPK1 kinase activity by the chemical compound necrostatin-1 or by knock-in of a kinase-dead form of RIPK1 can ameliorate necroptosis-associated pathologies in mouse models of various diseases, including inflammatory diseases (e.g. colitis and dermatitis) and in neurodegenerative diseases (e.g. multiple sclerosis (MS) and amyotrophic lateral sclerosis (ALS))^24,25^. Therefore, the suppression of the RIPK1 kinase activity could be a promising therapeutic approach for the treatment of these diseases. Recently, it has been shown that suppression of RIPK1 kinase activity improves anti-tumor immunity by modulating tumor-associated macrophages^26^; this occurs independently of RIPK3-mediated necroptosis, demonstrating that the kinase activity of RIPK1 can be viewed as a new immunomodulatory target for the development of new anti-cancer therapies.

The kinase domain of RIPK1 is thus an attractive target for intervention with specific chemical inhibitors. Necrostatin-1 (Nec-1) was identified as the first necroptosis inhibitor; it directly blocks RIPK1 kinase activity^17,27^. Nec-1 and its improved analog Nec-1s (7-Cl-O-Nec-1) were found to interact with RIPK1 at the back pocket of the ATP-binding site and to exhibit remarkable kinome selectivity^28–30^. However, these compounds displayed moderate potency, suboptimal pharmacokinetic profiles, and had off-target activities (e.g. indoleamine-2,3-dioxygenase). The RIPK1 inhibitors developed by GSK such as GSK963 and GSK2982772 have been shown to be extremely potent in human cells, but they have highly reduced cellular efficacy in mouse and rat^31,32^. This species selectivity imposes limitations on explorations of their in vivo therapeutic value with mouse or rat disease models. Therefore, identifying novel RIPK1 inhibitors with high efficacy and conserved potency among human, mouse, and rat would greatly facilitate the development of new therapeutic approaches. In the present study, we discovered PK68 as a novel inhibitor of RIPK1 kinase activity that displays potent cellular efficacy and that suppresses necroptosis in human, mouse, and rat cells. In addition, PK68 displays favorable kinome selectivity and pharmacokinetic properties. Notably, pre-treatment of PK68 powerfully blocks TNF-induced lethal shock and inflammatory responses as well as tumor metastasis in vivo. These findings suggest that PK68 can be used in the development of new therapies for the treatment of necroptosis-activated pathologies including inflammatory disorders and cancer metastasis.

## Results

### PK6 efficiently blocks TNF-α induced necroptosis in both human and mouse cells

**Fig. 1:**
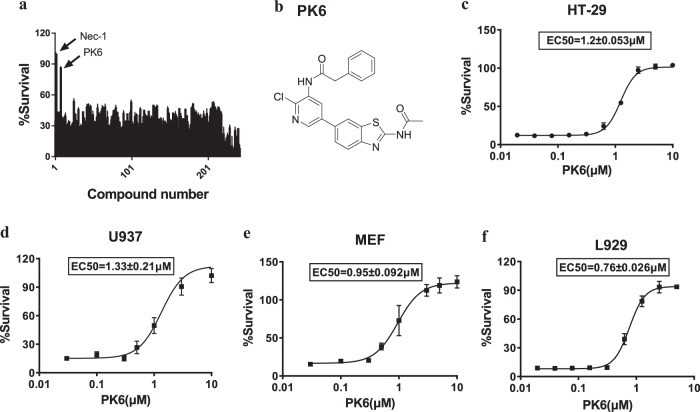
PK6 efficiently blocks TNF-α induced necroptosis in both human and mouse cells. **a** HT-29 cells cultured in 96-well plates were pretreated with the indicated compounds (10 μM) from a library consisting of ~240 compounds that we designed with novel structures for 1 h and subsequently treated with 40 ng/ml TNF-α (T), 100 nM Smac mimetic (S), and the caspase inhibitor 20 μM z-VAD(Z) for 48 h. Cell viability was assessed by measuring ATP levels. Data are represented as the mean ± standard deviation of duplicates. **b** Chemical structure of PK6. **c** Dose response curve and EC_50_ ± SD value for PK6 in TNF-α induced necroptosis in HT-29 cells. Cells were pretreated with indicated concentrations of PK6 for 1 h prior to the treatment with T (40 ng/ml), S (100 nM), and Z (20 μM) for 48 h. Cell viability was assessed by measuring ATP levels. **d** U937 cells were pretreated with indicated concentrations of PK6 for 1 h followed by the treatment with T (40 ng/ml), S (100 nM), and Z (20 μM) for 24 h. **e** MEF cells were pretreated with indicated concentrations of PK6 for 1 h followed by the treatment with T (40 ng/ml), S (100 nM), and Z (20 μM) for 24 h. **f** L929 cells were pretreated with indicated concentrations of PK6 for 1 h prior to the treatment with S (100 nM) and Z (20 μM) for 24 h. Data are represented as the mean ± standard deviation of triplicates

Necroptosis is tightly regulated by RIP kinases including RIPK1 and RIPK3. To develop new necroptosis inhibitors, we screened a small-molecule library of around 240 potential kinase inhibitors that were designed with novel structures to affect the ATP-binding pocket of protein kinases. Human colon cancer HT-29 cells were treated with these compounds for 2 h, followed by treatment with necroptotic stimuli (TNFα, Smac mimetic, and z-VAD) that are known to activate TNF-mediated necroptosis^2^. After 48 h, cell viability was analyzed by monitoring ATP levels. This screen identified PK6 as an effective inhibitor of TNF-induced necroptosis with an EC_50_ of ~1.2 μM (Fig. 1a–c). Consistently, PK6 blocked TNF-induced necroptosis in human leukemia U937 cells with an EC_50_ of ~1.33 μM (Fig. 1d). Further, we evaluated the effect of PK6 on TNF-induced necroptosis in mouse cells and found that PK6 efficiently inhibited TNF-induced necroptosis in both mouse embryonic fibroblasts (MEFs) with EC_50_ of ~0.95 μM and mouse fibroblast L929 cells with EC_50_ of ~0.76 μM (Fig. 1e, f). Taken together, these results demonstrate that PK6 is an inhibitor of TNF-induced necroptosis in both human and mouse cells.

### Chemical optimization of PK6 leads to improvements in anti-necroptotic activity

**Fig. 2:**
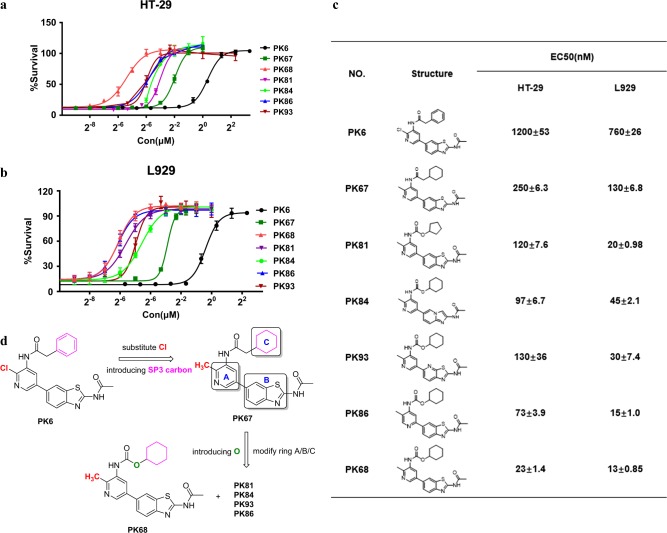
Chemical optimization of PK6 leads to improvements in anti-necroptotic activity. **a**, **b** The effects of the indicated compounds on cell viability were examined in HT-29 cells (**a**) and L929 cells (**b**). HT-29 cells were treated as indicate for 48 h and L929 cells for 24 h. Cell viability was assessed by measuring ATP levels. Data are represented as the mean ± standard deviation of triplicates. **c** Structure and EC_50_ ± SD value of each compound are shown in the table. EC_50_ values are the average of at least two independent determinations. **d** Lead optimization of PK6

Having established that PK6 is a novel inhibitor of TNF-induced necroptosis in both human and mouse cells, we next explored strategies to enhance the anti-necroptotic efficacy and drug-like properties of PK6. As a screening hit, PK6 exhibited numerous undesirable characteristics, for example the 2-chlorine atom on the center pyridine ring might act as an active leaving group, thereby potentially eliciting toxicity in biological systems (Fig. 2d, marked in red). Furthermore, the solubility of PK6 was poor, most likely due to the number of sp2 carbons present in PK6.

In order to address these problems, we used a bioisosteric strategy^33^ to substitute the potentially toxic chlorine with a methyl group^34^; we also introduced sp3 carbons on ring C (Fig. 2d, marked in pink)^35^. The results were very encouraging: PK67 displayed significant improvement in inhibiting TNF-induced necroptosis in both human and mouse cells (250 and 130 nM, respectively; Fig. 2a–c). Further modifications were made on the center ring A and the bicyclic ring B, and an oxygen atom was introduced at the top region of the scaffold (Fig. 2d, marked in green). These modifications proved to be successful: numerous compounds with improved potency were thusly obtained (e.g., PK81, 84, 86, 93; Fig. 2a–c). These efforts ultimately culminated in the identification of PK68, a highly potent inhibitor of TNF-induced necroptosis (23 and 13 nM in human and mouse cells, respectively; Fig. 2a–c). PK68 exhibits significant improvement in anti-necroptotic activity and in its physicochemical properties as compared with the screening hit PK6 (Supplementary Table 1). The structural evolution underlying the development of PK68 is summarized in Fig. 2d. The synthetic experimental details and characterization of compounds can be found in supplementary information (Supplementary Figs. 1–18 and Synthetic Procedures).

### PK6 and PK68 block cellular activation of RIPK1, RIPK3, and MLKL upon necroptotic stimuli

**Fig. 3:**
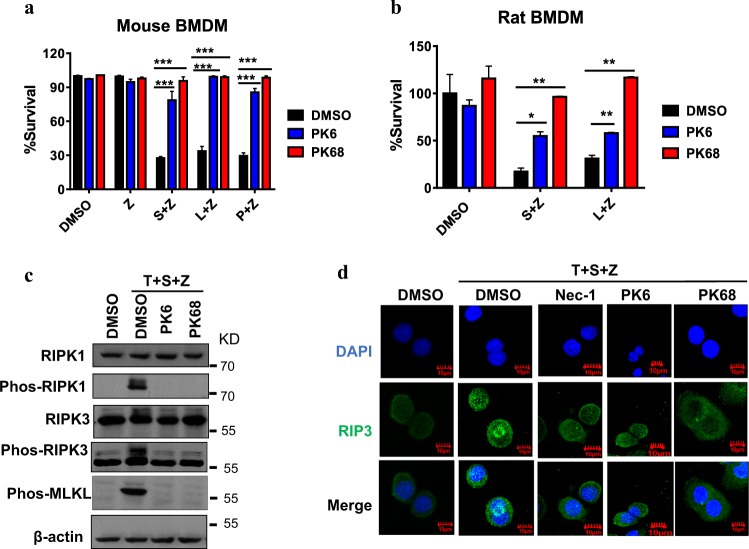
PK6 and PK68 block cellular activation of RIPK1, RIPK3, and MLKL upon necroptotic stimuli. **a**, **b** Bone marrow-derived macrophages from C57BL/6 mice or rat were pretreated with DMSO, PK6(10 μM), or PK68 (100 nM) for 1 h and subsequently treated with indicated stimuli for 24–30 h. Cell viability was determined by measuring ATP levels. L, LPS (20 ng/ml); P, poly(I:C) (50 μg/ml); S (100 nM); Z (10 μM). Data are represented as the mean ± standard deviation of triplicates. **c** HT-29 cells were treated with the indicated compound for 1 h prior to the treatment of T (40 ng/ml), S (100 nM), and Z (20 μM) for additional 8 h. Cell lysates were harvested and subjected to western blot analysis for phosphorylation of RIPK1, RIPK3, and MLKL. **d** The effects of PK6 and PK68 on the formation of RIPK3 puncta. HT-29 cells stably expressing Flag-tagged RIPK3 were pretreated with indicated compounds for 1 h prior to the treatment of T (100 ng/ml), S (100 nM), and Z (20 μM) for additional 12 h. The distribution of RIPK3 was detected by immunofluorescence. *P < 0.05. **P < 0.01. ***P < 0.001

In addition to TNF-induced necroptosis, it is known that TLR3 or TLR4 activation can also trigger necroptosis^36^. We further evaluated the effects of PK6 and PK68 on TLR3- and TLR4-mediated necroptosis induced by poly(I:C)/z-VAD and LPS/z-VAD, respectively. Treatment of PK6 or PK68 significantly blocked both TLR3- and TLR4-mediated necroptosis in mouse bone marrow-derived macrophages (Fig. 3a). Consistently, in rat bone marrow-derived macrophages, PK6 and PK68 showed inhibitory effects on both TNFR- and TLR-mediated necroptosis (Fig. 3b). These results support that PK6 and PK68 have a common mechanism of inhibiting the conserved necroptosis signaling pathways among mouse, rat, and humans. To investigate the molecular mechanism of PK series-mediated necroptosis inhibition, we examined the effects of these compounds on the major steps of necroptotic signaling, including activation of RIPK1, RIPK3, and MLKL. RIPK1, RIPK3, and MLKL were phosphorylated upon necroptotic stimuli, while addition of PK6 or PK68 completely abolished phosphorylation of RIPK1, RIPK3, and MLKL (Fig. 3c). It has been shown previously that RIPK3 activation leads to the formation of RIPK3 puncta during necroptosis. We observed that treatment of PK6 or PK68 prevented generation of RIPK3 puncta (Fig. 3d), suggesting that PK6 and PK68 block necroptosis through the suppression of RIPK3 function or signaling upstream of RIPK3 activation.

### PK68 is a highly selective inhibitor of RIPK1 kinase activity

**Fig. 4:**
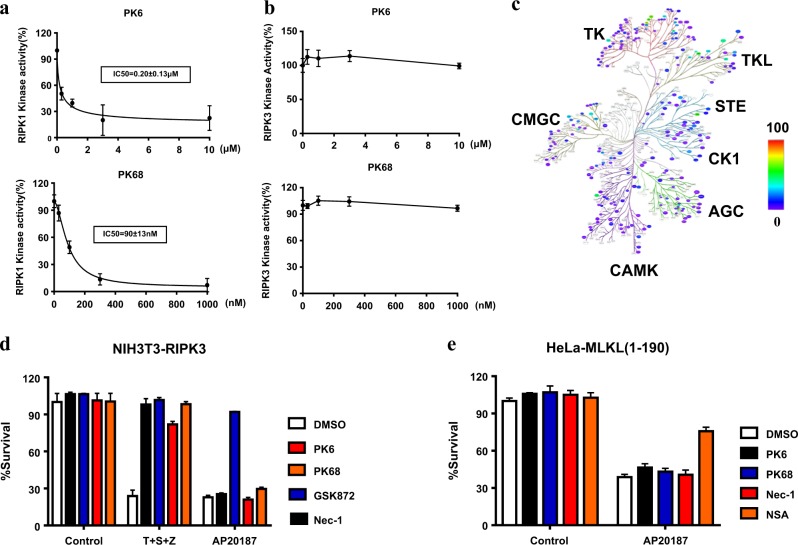
PK68 is a highly selective inhibitor of RIPK1 kinase activity. **a** In vitro kinase activity assays using recombinant RIPK1 (**a**) and RIPK3 (**b**) were performed. Recombinant proteins were incubated with PK6 and PK68 as indicated. Data represent mean value ± standard deviation. **c** PK68 shows good kinase selectivity against a panel of 369 kinases (without RIPK1) provided by Reaction Biology as viewed in the human kinome phylogenetic tree. **d** PK6 and PK68 inhibit TNF-induced necroptosis but not RIPK3 dimerization-induced cell death in NIH3T3-RIPK3 cells that stably express RIPK3 fused to FKBP F36V mutant. NIH3T3-RIPK3 cells were pretreated with indicated compounds for 1 h and subsequently treated with T (40 ng/ml)/S (100 nM)/Z (20 μM) or AP20187 (100 nM) for 24 h. Cell viability was determined by measuring ATP levels. **e** PK6 and PK68 have no effect on MLKL dimerization-induced cell death in HeLa-MLKL (1–190) cells that stably express MLKL (1–190aa) fused to DmrB. HeLa-MLKL (1–190) cells were pretreated with indicated compounds for 1 h and subsequently treated with AP20187 (100 nM) for 24 h. Cell viability was determined by measuring ATP levels. Data are represented as the mean ± standard deviation of triplicates

Having shown that PK6 and PK68 can inhibit activation of both RIPK1 and RIPK3 during TNF-mediated necroptosis, we sought to determine if PK6 and PK68 can directly target RIPK1 or and RIPK3 by performing in vitro kinase assays. Both PK6 and PK68 were able to block the kinase activities of both human RIPK1 and mouse RIPK1 in vitro (Fig. 4a and Supplementary Fig. 19). Compared to PK6, PK68 showed improved activity for inhibiting RIPK1 kinase activity, with an IC_50_ of around 90 nM, a value consistent with its cellular activity against necroptosis. In contrast, neither PK6 nor PK68 affected RIPK3 kinase activity, even at a 1000 nM concentration (Fig. 4b).

Further, we evaluated the selectivity of PK68 against a panel of 369 kinases provided by Reaction Biology (at 1000 nM). PK68 was found to be a reasonably selective RIPK1 inhibitor, displaying >50% inhibition of five kinases (TRKA, TRKB, TRKC, TNIK, and LIMK2) among all of the other tested kinases (Fig. 4c and Supplementary Table 2). In addition, we followed on some of the active “hits” from the kinome scan. PK68 was tested against TNIK and TRKA by a reputable CRO company. PK68 was found to be not very active against these two kinases, with IC_50_ ~10,000 nM (Supplementary Fig. 20). RIPK1 is known to mediate NF-κB activation independently of its kinase activity^23^. Consistently, PK6 and its derivatives including PK68 showed no impact on TNF-induced NF-κB activation (Supplementary Fig. 21). It has been shown that homodimerization of RIPK3 or MLKL induced by the dimerizer leads to necroptosis bypassing the upstream signals^10,37^. We conducted experiments in which NIH3T3 cells stably expressing mouse RIPK3 fused to FK506-binding protein (FKBP) carrying the F36V mutant were treated with the dimerizer AP20187 to enforce dimerization of RIPK3, and found that this successfully led to the activation of RIPK3 and necroptosis (Fig. 4d). This dimerized RIPK3-induced necroptosis was blocked by RIPK3 inhibitor GSK873 but was not blocked by the RIPK1 inhibitor necrostatin-1. Moreover, neither PK6 nor PK68 blocked RIPK3 dimerization-induced necroptosis (Fig. 4d), suggesting that both PK6 and PK68 do not affect RIPK3 or its downstream events. HeLa expressing MLKL (1–190aa) fused to DmrB, which is identical to FKBP F36V mutant, treated with AP20187 resulted in MLKL polymerization, which has been reported to activate MLKL and necroptosis independently of RIPK1 and RIPK3^38^ (Fig. 4e). Consistently, neither PK6 nor PK68 had any effect on this polymerized MLKL-induced form of necroptosis, which was however effectively suppressed by MLKL inhibitor necrosulfonamide (NSA) (Fig. 4e). Collectively, these results demonstrate that PK68 is a potent and selective inhibitor of necroptosis that acts by suppressing RIPK1 kinase activity.

### A docking study indicates that PK68 is a type II inhibitor of RIP1 kinase

**Fig. 5:**
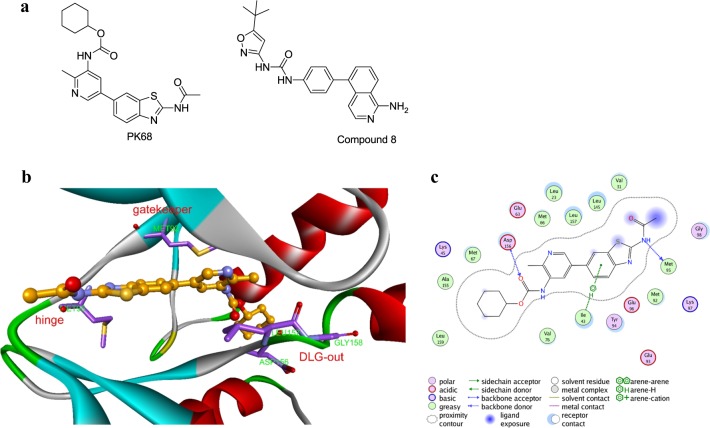
The molecular docking of PK68 on RIPK1 indicates PK68 as a type II inhibitor of RIP1 kinase. **a** Chemical structures of PK68 and compound 8 in 4NEU. **b**The predicted binding conformation of PK68 derived from Glide docking study. **c** Schematic representation of the interaction patterns between PK68 and the key residues in the binding pocket of RIPK1 kinase

To analyze the interaction patterns between PK68 and RIPK1 kinase, we docked PK68 into the binding pocket of the RIPK1 crystal structure (PDB ID: 4NEU^39^) using the extra precision (XP) scoring function in *Glide* docking^40^. Note that detailed descriptions of binding site generation and the *Glide* docking pipeline have been described in our previous study^41^. The chemical structures of PK68 and compound 8 from 4NEU are shown in Fig. 5a. The predicted binding conformation of PK68 and the interaction patterns between PK68 and RIPK1 kinase domain are shown in Fig. 5b and c, respectively.

Similar to the co-crystallized ligand of the 4NEU crystal complex, PK68 was predicted as a typical type II kinase inhibitor; it interacted with a DLG (Asp156–Leu157–Gly158)-out form of the RIPK1 protein (Fig. 5b). The N-acetamide of PK68 is apparently a hinge binder, forming hydrogen bond interaction with the backbone CO of residue Met95. The *benzo[d]thiazole* in the tail group (*N-(benzo[d]thiazol-2-yl)acetamide*) of PK68 can interact with residue Ile43 (arene-H interaction) (Fig. 5c). In addition, the *carbonyl oxygen* of *carbamic acid* in the head group of PK68 can form a hydrogen bond with the backbone amide of residue Asp156 in the DLG motif. Moreover, the *cyclohexane* group of PK68 is buried deeply in the hydrophobic allosteric pocket that encompasses residues Met66, Met67, Leu70, Val75, Leu129, Val134, and Leu159^39^ created by the DLG-out conformation in RIPK1 (Fig. 5b, c).

### PK68 exhibits a favorable pharmacokinetic profile and no obvious toxicity in mice

**Fig. 6:**
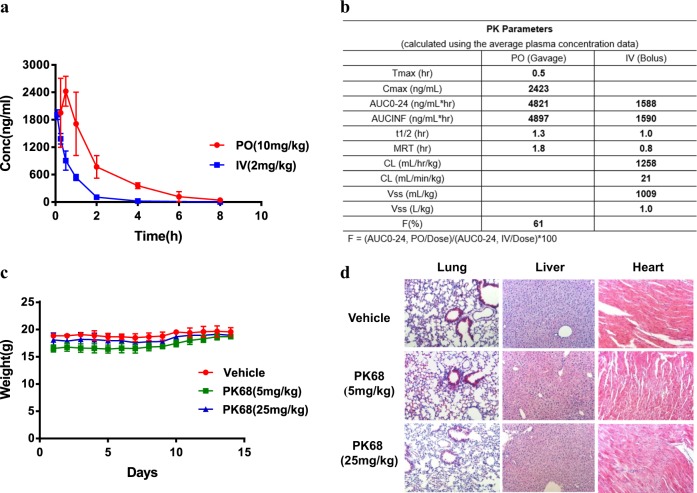
PK68 exhibits a favorable pharmacokinetic profile and no obvious toxicity in mice. **a** Plasma concentration of PK68 versus time curves for peros (PO) and intravenous injection (IV). Data represent mean value ± standard deviation. **b** Plasma pharmacokinetic parameters of PO and IV. **c**, **d** C57BL/6 mice (*n* = 5) were treated with the indicated daily oral gavage dosing of PK68 for successive 14 days. Body weight (**c**) and hematoxylin-eosin (H&E) staining (**d**) of mice were analyzed. Control mice received an equal amount of vehicle. Magnification: ×40. Data represent mean value ± standard deviation

Encouraged by our overall satisfactory in vitro potency and selectivity data for PK68, we decided to assess its in vivo pharmacokinetic profile. When dosed orally in ICR mice, PK68 was quickly absorbed into the bloodstream with a Tmax of 0.5 h and a Cmax of 2423 ng/ml. PK68 displayed a moderate clearance (21 ml/min/kg), a good steady-state volume of 1.0 L/kg, and a half-life of 1.3 h. The oral exposure of PK68 was good, with an AUC of 4897 ng h/ml, leading to an estimated oral bioavailability of 61% (Fig. 6a, b).

PK68 exhibited an EC_50_ ~20 nM in the cell-based assay (HT-29 cells treated with TNFα/Smac mimetic/z-VAD). When dosed orally at 10 mg/kg in mice, PK68 exhibited a Cmax of 2423 ng/ml, translating to a plasma concentration of ~4000 nM. Judging by the PK time curve, the observed plasma levels of PK68 were above its EC_50_ over a period of 8 h. Based on these results, we hypothesized that the preliminary toxicity of PK68 could be evaluated at a low dose (5 mg/kg) and a high dose (25 mg/kg, which should support plasma concentration of PK68 well above its EC_50_ for 24 h). These doses were meant to determine the preliminary toxicity effects of PK68 (Fig. 6c). PK68 was well tolerated at both doses (5 and 25 mg/kg, daily oral gavage dosing). All tested animals exhibited normal body weight and behavior, and consumed a normal amount of food as compared with the vehicle group throughout the 14-day observation period (Fig. 6c). We further performed histology of various tissues from mice that had received 14-day treatment of PK68. Tissues including lung, liver, and heart from PK68-treated mice showed normal morphology like the vehicle-treated mice (Fig. 6d). Collectively, these results show that PK68 exhibits favorable pharmacokinetic profiles and no obvious toxicity in mice treated with a 14-day course at a dose of 25 mg/kg.

### PK68 ameliorates TNF-induced SIRS

**Fig. 7:**
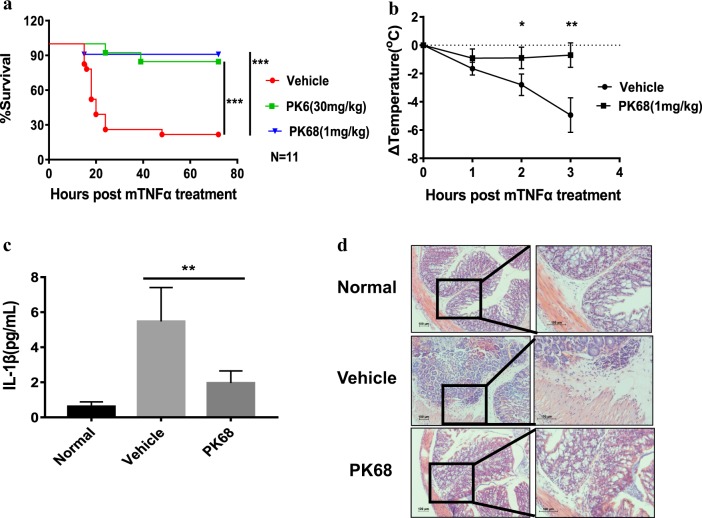
PK68 ameliorates TNF-induced systemic inflammatory response syndrome. C57BL/6 mice (*n* = 11) were injected with vehicle, PK6 (30 mg/kg), or PK68 (1 mg/kg) via intraperitoneal injection for 15 min, followed by the tail intravenous injection of mouse TNF-α (6 μg per mouse). **a**, **b** The survival rate (**a**) and body temperature loss (**b**) were monitored. **c**, **d** C57BL/6 mice (*n* = 5) were injected with vehicle or PK68 (1 mg/kg) for 15 min prior to the injection of mouse TNF-α. The mice were sacrificed 4 h after TNF-α administration, the serum concentration of IL-1β was measured by ELISA kits (**c**), and histology of the colon tissue was analyzed by H&E staining and the representative images are shown (**d**). The serum or colon tissue from C57BL/6 mice was used as the control (Normal). Magnification: ×100 (left) and ×200 (right). Data represent mean value ± standard deviation. *P < 0.05. **P < 0.01. ***P < 0.001

Necroptosis is involved in a variety of inflammatory disorders such as TNF-induced SIRS^11,12^. We therefore evaluated the therapeutic effect of PK68 in a mouse model of TNF-induced SIRS. C57BL/6 mice were treated with vehicle, PK6, or PK68 for 15 min prior to intravenous injection of mouse TNFα (6 µg TNFα per mouse). Treatment with 30 mg/kg PK6 or 1 mg/kg PK68 provided effective protection against TNFα-induced lethal shock (Fig. 7a). Consistently, treatment of PK68 strongly reduced TNFα-induced temperature loss in mice (Fig. 7b). SIRS is associated with systemic release of proinflammatory cytokines and tissue injuries^11^. In TNF-induced SIRS, PK68 treatment significantly ameliorated the production of proinflammatory cytokines including IL-1β (Fig. 7c). Moreover, TNF-induced damage of the colon was attenuated by the treatment of PK68 (Fig. 7d). Taken together, these results demonstrate that inhibition of RIPK1 by PK68 provides strong protection against TNF-induced SIRS, thus highlighting PK68 as an RIPK1 inhibitor with very promising potential for the development of anti-inflammatory therapeutics.

### PK68 displays preventive suppression of tumor metastasis in the mouse cancer models

**Fig. 8:**
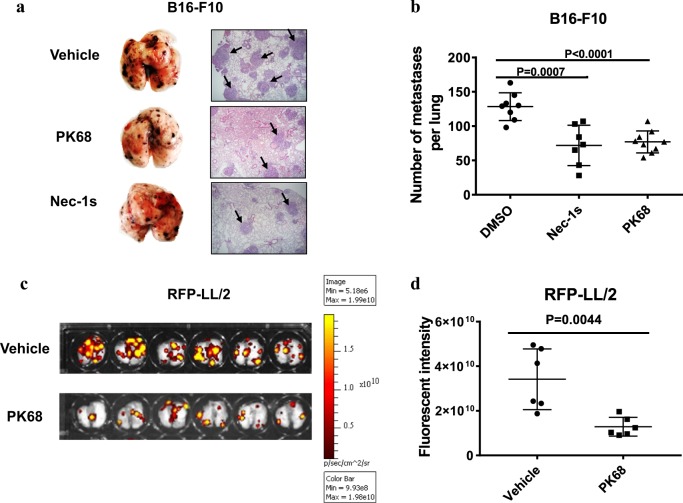
PK68 significantly represses tumor metastasis in the mouse cancer models. C57BL/6 mice bearing B16-F10 cells (**a**, **b**) or RFP-labeled LL/2 cells (**c, d**) were treated with vehicle, PK68 (5 mg/kg) or Nec-1s (5 mg/kg) as described in the Materials and methods. **a**, **b** Tumor metastasis of B16-F10 was measured by the numbers of metastasis nodules and histological analysis. Representative images of the lungs and lung section staining by H&E staining (**a**). The arrowhead indicates tumor cells. Magnification: ×40. The numbers of metastasis nodules on lung surfaces (**b**) in the metastasis model of B16-F10 were counted with the microscope. **c**, **d** Tumor metastasis of RFP-labeled LL/2 was calculated by RFP luciferase intensity. Representative pictures and statistical diagram are present. Data represent mean value ± standard deviation

Studies have shown that RIPK1 kinase activity is involved in the promotion of tumor metastasis through death receptor 6-dependent necroptosis of endothelial cells^22^ or VEGF-dependent activation of vascular permeability^42^. We thus evaluated the therapeutic effect of PK68 on lung metastasis of mouse melanoma B16-F10 cells in vivo. As shown in Fig. 8a, C57BL/6 mice received tail intravenous injection of B16-F10 cells and then developed lung metastasis. After 2 weeks, the metastasis of B16-F10 cells was measured by histological analysis and assessed as the number of metastasis nodules in the lung. Notably, pre-treatment of PK68 significantly reduced the number of pulmonary metastasis nodules, suggesting an inhibitory effect of PK68 on metastasis of B16-F10 cells to the lungs in mice (Fig. 8a, b). Consistently, PK68-pre-treated mice showed markedly decreased lung metastasis of RFP-labeled Lewis lung carcinoma LL/2 (RFP-LL/2) cells compared to vehicle-treated mice (Fig. 8c, d). Both B16-F10 and RFP-LL/2 cells express RIPK1, but they do not have RIPK3 (Supplementary Fig. 22). Two weeks after intravenous injection of RFP-LL/2 cells, RFP^+^ LL/2 cells were isolated from the tumor tissues by flow cytometer sorting and no signal of phosphorylated RIPK1 was detected in these tumors cells (Supplementary Fig. 22). Six hours after intravenous injection of RFP-LL/2 cells into C57BL/6 mice, the RFP-LL/2 cells have extravasated through blood vessels into the lung (Supplementary Fig. 23). We found that pre-treatment of PK68 resulted in decreased number of RFP-LL/2 cells in the lung 6 h after intravenous injection of RFP-LL/2 cells (Supplementary Fig. 23), supporting an inhibitory effect of PK68 on extravasation of tumor cells through blood vessels. Further, we evaluated the effect of PK68 on the tumor cell transmigration across the cultured endothelium. We isolated CD31^+^ endothelial cells from the lungs and performed the transendothelial migration assay as previously reported^42^. Addition of PK68 attenuated transmigration of RFP-LL/2 cells through the endothelial cell monolayer (Supplementary Fig. 24), while treatment of PK68 or Nec-1s had no obvious influence on the proliferation rate and invasion ability of B16-F10 or RFP-LL/2 cells without the endothelial cell monolayer in vitro (Supplementary Fig. 25). These findings demonstrate that inhibition of RIPK1 by PK68 results in attenuated tumor cell transmigration across the endothelial barrier and preventive suppression of tumor metastasis.

## Discussion

Necroptosis is integral to the pathogenesis of conditions including acute and chronic inflammatory disorders, neuronal and renal degeneration, tumor metastasis, and ischemia–reperfusion-induced tissue injury. Therefore, the essential regulators of the necroptosis signaling pathway like RIPK1 are viewed as promising therapeutic targets. In the current study, we discovered that PK6 and its optimized derivatives including PK68 are a class of novel necroptosis inhibitors that can highly selectively suppress RIPK1 kinase activity and potently block necroptotic cell death, both in vitro and in vivo.

RIPK1 belongs to the RIP kinase family comprising the seven members RIPK1–RIPK7. RIPK1 regulates necroptosis via its kinase function, and it also mediates NF-κB activation as an adaptor protein independently of its kinase activity^5,23^. NF-κB signaling plays is essential for the regulation of multiple inflammatory responses, for cell proliferation, and cell survival^43^. Genetic studies have shown that loss of RIPK1 in mice results in lethality after birth, while mice expressing an RIPK1 kinase dead mutant variant are viable and develop normally^12,44^. Thus, inhibition of RIPK1 kinase activity can be viewed as a potentially attractive therapeutic strategy for intervention in necroptosis without the need to alter its function in the NF-kB pathway. We designed around ~300 small molecules as potential kinase inhibitors with novel structures. Among these, PK6 was identified as an inhibitor of necroptosis using a cell-based assay. Structural optimization of PK6 led to the discovery of PK68, which displayed potent cellular efficacy and in vitro inhibition of RIPK1 kinase activity, with an IC_50_ of ~90 nM.

Assessment of the predicted binding mode demonstrated that PK68 bound to the ATP domain with a DLG-out configuration, suggesting PK68 as a type II kinase inhibitor. This result is consistent with the notion that PK68 specifically blocks the kinase activity of RIPK1, but does not interfere with other RIP family members including RIPK2, RIPK3, RIPK4, or RIPK5. Notably, an analysis of 369 human kinases revealed reasonable selectivity of PK68 for RIPK1. As a bona fide RIPK1 kinase inhibitor, PK68 displayed potent cellular efficacy in inhibiting necroptosis cross mice, rat, and humans. This is in contrast to GSK2982772, a type III allosteric RIPK1 inhibitor, which displayed significant differences in activity amongst species. Furthermore, RIPK1 is a pleiotropic protein capable of mediating cell death and NF-κB signaling. RIPK1 kinase inhibitors, especially those bind to allosteric sites, might induce conformational changes in the protein and therefore impact the scaffolding function of RIPK1 and the ripoptosome, a RIPK1-containing signaling platform for inducing cell death^45^. Supporting this idea, our RIPK1 inhibitors including PK6 and PK68 had no effect on NF-κB activation (Supplementary Fig. 21).

When evaluated in vivo, PK68 displayed a favorable pharmacokinetic profile with an oral bioavailability of 61% and no detectable toxicity in mice. Furthermore, PK68 is a potent agent against TNF-induced lethal shock, tissue damage, and induction of inflammatory cytokines. Importantly, PK68 significantly repressed tumor metastasis in the mouse cancer models of melanoma and lung carcinoma. These findings strongly highlight the great potential of PK68 for the development of novel anti-inflammatory and anti-metastasis therapies. It is also worth noting that the kinase activity of RIPK1 is required to mediate TNF-induced apoptosis in the absence of the activities of cellular inhibitor of apoptosis proteins (cIAPs)^46–48^. In addition, inhibition of RIPK1 kinase activity has been found to attenuate the production of inflammatory cytokines and to improve anti-tumor immunity independently of its role in cell death. Therefore, further understanding of the precise role of RIPK1 kinase activity in relevant diseases models will be critical for the development of valuable therapies using RIPK1 inhibitors.

## Materials and methods

### Cells and reagents

HT-29 cells, U937 cells, MKN45 cells, L929 cells, and Panc-1 cells were from ATCC. HT-29-RIPK3 cells, NIH3T3-RIPK3 cells, and mouse MEFs were kindly provided by Dr. Xiaodong Wang (National Institute of Biological Sciences (NIBS), Beijing). HeLa-MLKL (1–190) cell line was a gift from Dr. Zhigao Wang (University of Texas Southwestern Medical Center at Dallas). These cells were cultured in Dulbecco’s modified Eagle’s medium (Hyclone) supplemented with 10% fetal bovine serum (Invitrogen) and 2 mM l-glutamine (Invitrogen). Bone marrow-derived macrophages were isolated from the bone marrow of ~7-week-old mice or ~25-month-old rats and cultured for 7 days in the medium containing 30% L929-cell-conditioned medium, 20% fetal bovine serum (FBS), and 50% RPMI-1640. L929-cell-conditioned medium containing colony-stimulating factor was collected after growing L929 cells in Dulbecco's modified Eagle's medium (DMEM) plus 10% FBS for 7–10 days as previously described^36^.

The Smac mimetic compound was kindly provided by Dr. Xiaodong Wang (National Institute of Biological Sciences, Beijing). Necrostatin-1 and z-VAD were purchased from Alexis Biochemicals and Bachem, respectively. LPS and Poly(I:C) were from Sigma and invivoGen, respectively. Recombinant mouse TNF-α was purchased from Genscript. Human recombinant RIPK1, RIPK3, and mouse recombinant RIPK1 were purchased from SignalChem. The cellTiter-Glo Luminescent cell viability assay kit and ADP-Glo kinase assay kit were purchased from Promega.

### Antibodies

The following antibodies were used: anti-RIPK1 monoclonal antibody (BD Bioscience, 610459), anti-phospho-RIPK1 monoclonal antibody (Cell Signaling, #65746), anti-Flag monoclonal antibody (Sigma-Aldrich, F3165), anti-mouse-RIPK3 (Prosci, 2283), anti-phospho-IκB-α monoclonal antibody (Cell Signaling, 9246), and anti-β-actin monoclonal antibody (Sigma-Aldrich, A2066). The anti-human-RIPK3 polyclonal antibody was generated against full-length human RIPK3 recombination protein.

### Cell survival assay

The cell viability was analyzed by using the CellTiter-Glo Luminescent Cell Viability Assay kit following the manufacturer’s instructions (Promega). Luminescence was measured with SpectraMax i3x (Molecular Devices).

### Western blot analysis

Cell pellet was collected by centrifugation at 13000 × *g* for 1 min and resuspended in lysis buffer (20 mM Tris-HCl, pH 7.4, 150 m1M NaCl, 10% glycerol, 1% Triton X-100, 1 mM Na_3_VO_4_, 25 mM β-glycerol phosphate, 0.1 mM PMSF, a complete protease inhibitor set (Roche)). The resuspended cell pellet was lysed on ice for 20 min. Then, cell lysates were centrifuged at 13000 × *g* for 20 min at 4 ℃. The supernatants were collected and subjected to western blot analysis.

### Immunofluorescent staining

HT-29 expressing Flag-RIP3 cells were seeded in a chamber slide and cultured overnight. These cells were pretreated with indicated compounds for 1 h, followed by treatment with TNF-α, Smac mimetic, and z-VAD for 12 h. The cells were then washed with phosphate-buffered saline (PBS) followed by fixation in 4% paraformaldehyde for 10 min. The cells were further washed three times with PBS followed by incubation with 0.25% Triton X-100 in PBS for 10 min. After that, cells were blocked for 30 min with 5% BSA in PBS and stained with anti-flag antibody and secondary antibody successively. Nuclei was stained with DAPI. Images were captured with a Olympus confocal microscope.

### In vitro kinase activity assay

The recombinant RIPK1 or RIPK3 protein was incubated with DMSO or the indicated compound for 15 min in the assay buffer (25 mM HEPES pH 7.2, 20 mM MgCl_2_, 12.5 mM MnCl_2_, 12.5 mM β-glycerol phosphate, 5 mM EGTA, 2 mM EDTA, and 2 mM DTT). Then, ATP (50 μM) and the substrate MBP (20 μM) were added to the reaction at room temperature for 120 min. The luminescence was measured to calculate the kinase activity after the addition of the ADP-Glo Kinase Assay kit following the manufacturer’s instructions (Promega).

### Kinase selectivity profile

PK68 was tested at 1 μM in duplicate against a panel of 369 human kinases at Reaction Biology Corporation. Results are viewed in the human kinome phylogenetic tree.

### Source of animals

C57BL/6 male mice were purchased from Beijing Vital River Laboratory Animal Technology Co., Ltd. All mice were bred under standard conditions and used at the age of 6–8 weeks with about 20 g body weight. All animal experiments were approved by the Soochow University Ethics Committee.

### TNF-induced SIRS

PK6 and PK68 were diluted into sterile PBS. C57BL/6 mice were pretreated with vehicle, PK6 (30 mg/kg), or PK68 (1 mg/kg) via intraperitoneal injection for around 15 min and then challenged with mouse TNF-α (6 μg per mouse) via tail intravenous injection. Mice mortality was continuously monitored till 72 h after TNF-α administration. The serum and colon tissue are collected at 4 h post TNF-α challenge.

### The mouse models of tumor metastasis

Melanoma B16-F10 cells (0.5 × 10^6^ cells) or RFP-labeled lung carcinoma LL/2 cells (0.3 × 10^6^ cells) were injected into C57BL/6 mice in a volume of 200 μl PBS via the tail intravenous injection. Vehicle, PK68 (5 mg/kg), or Nec-1s (5 mg/kg) was injected intraperitoneally to these mice 30 min before tumor cells injection, 3 and 6 h after tumor cells injection. Then, these mice were given the same dose of vehicle, PK68, or Nec-1s every day for 7 days. Fourteen days after injection of tumor cells, mice were sacrificed and lung tissues were collected. The lung metastasis of B16-F10 cells in mice was determined by accounting the number of metastasis nodules with a dissecting microscope (Leica) and histologic analysis. The lung metastasis of LL/2 cells in mice was analyzed by measuring RFP fluorescence intensities with the PerkinElmer IVIS Lumina II. For evaluation of the number of extravasated tumor cells, lung tissues were harvested 6 h after intravenous injection of RFP-LL/2. Early metastatic RFP-LL/2 cells in the lung were viewed on a fluorescent microscope, and quantified by Image J software. Total RNA of lung tissues was extracted, and the expression level of RFP was analyzed by quantitative real-time PCR.

### Transendothelial migration assay

Primary CD31^+^ endothelial cells isolated from lungs by using CD31 beads (Miltenyi Biotec) were used for the transendothelial migration assay as previously described^42^. In brief, CD31^+^ endothelial cells were cultured for 5–6 days and then seeded on gelatin coated insert in EBM-2 (Lonza). After 2 days, cell culture media was changed with DMEM plus 5% FBS in the “bottom” wells and with DMEM plus 1% FBS in the “insert” wells, followed by the treatment of vehicle or PK68 for 2 h prior to addition of RFP-LL/2 tumor cells. After 20 h, the remaining cells in the “insert” wells were removed. Then fluorescence picture of transmigrated RFP-LL/2 cells were taken at ×40 magnification under the microscope (Nikon), and the intensity of fluorescence was analyzed by Image J software.

### Statistical analyses

Data of cell survival rate are represented as the mean ± standard deviation of duplicates or triplicates. All experiments were repeated at least three times with similar results. Significance was evaluated using *t-*tests of GraphPad Prism software.

## Supplementary information

Discovery of potent necroptosis inhibitors targeting RIPK1 kinase activity for the treatment of inflammatory disorder and cancer metastasis
