# Supplementary material for: Discovery of potent necroptosis inhibitors targeting RIPK1 kinase activity for the treatment of inflammatory disorder and cancer metastasis
Source: Cell Death Dis. 2019 Jun 24;10(7):493. doi: 10.1038/s41419-019-1735-6 (PMC6591251; doi:10.1038/s41419-019-1735-6)
Supplement: Supplementary file 1 — Discovery of potent necroptosis inhibitors targeting RIPK1 kinase activity for the treatment of inflammatory disorder and cancer metastasis [file 41419_2019_1735_MOESM1_ESM.docx]

**Discovery of potent necroptosis inhibitors targeting RIPK1 kinase activity for the treatment of inflammatory disorder and cancer metastasis**

Jue Hou^1,2#^, Jie Ju^1,2#^, Zili Zhang^1,2#^, Cong Zhao^1,2^, Zhanhui Li^3^, Jiyue Zheng^3^, Tian Sheng^3^, Hongijian Zhang^3^, Linkun Hu^5^, Xiaoliang Yu^1,2,4^, Wei Zhang^1,2,4^, Yangxin Li^6^, Meng Wu^1,2^, Haikuo Ma^1,3^*, Xiaohu Zhang^3^*, Sudan He^1,2,4^*

^1^Cyrus Tang Hematology Center and Collaborative Innovation Center of Hematology, State Key Laboratory of Radiation Medicine and Protection, Soochow University, Suzhou, Jiangsu, 215123, China

^2^ Key Laboratory of Stem Cells and Biomedical Materials of Jiangsu Province and Chinese Ministry of Science and Technology, Soochow University, Suzhou, Jiangsu, 215123, China

^3^Jiangsu Key Laboratory of Neuropsychiatric Diseases and College of Pharmaceutical Sciences, Soochow University, Suzhou, Jiangsu, 215123, China

^4^Center of Systems Medicine, Institute of Basic Medical Sciences, Chinese Academy of Medical Sciences & Peking Union Medial College, Beijing; Suzhou Institute of Systems Medicine, Suzhou, 215123 Jiangsu, China

^5^Department of Urology, The First Affiliated Hospital of Soochow University, 188 Shizi Rd, Suzhou 215006, China.

^6^Institute for Cardiovascular Science and Department of Cardiovascular Surgery, First Affiliated Hospital of Soochow University, Suzhou, Jiangsu, China.

^#^These authors equally contributed to this study.

* Corresponding authors:

Dr. Haikuo Ma（Email: mahaikuo123@163.com）or Dr. Xiaohu Zhang (Email: xiaohuzhang@suda.edu.cn), or Dr. Sudan He (Email: hesudan2018@163.com), Cyrus Tang Hematology Center and Collaborative Innovation Center of Hematology, Soochow University, 199 Ren'ai Rd, Suzhou, 215123, China. [Tel:86-512- 62875015](Tel:86-512-65880497)

**Supplementary Information**

|  | **page** |
| --- | --- |
| **Supplementary Figure S1-S25** | S3-S27 |
| **Supplementary Table S1, S2** | S28-S35 |
| **Detailed Synthetic Procedures** | S36-S45 |

**Supplementary Figure 1**: Synthetic schemes for **PK6**, **PK67**, **PK68**, **PK81** and **PK84** A) Synthesis of **PK6**, **PK67** and **PK81**; B) Synthesis of **PK68** and **PK84**.

**Supplementary Figure 2**: Synthetic schemes for **PK86** and **PK93**. C) Synthesis of **PK86**; D) Synthesis of **PK93**.


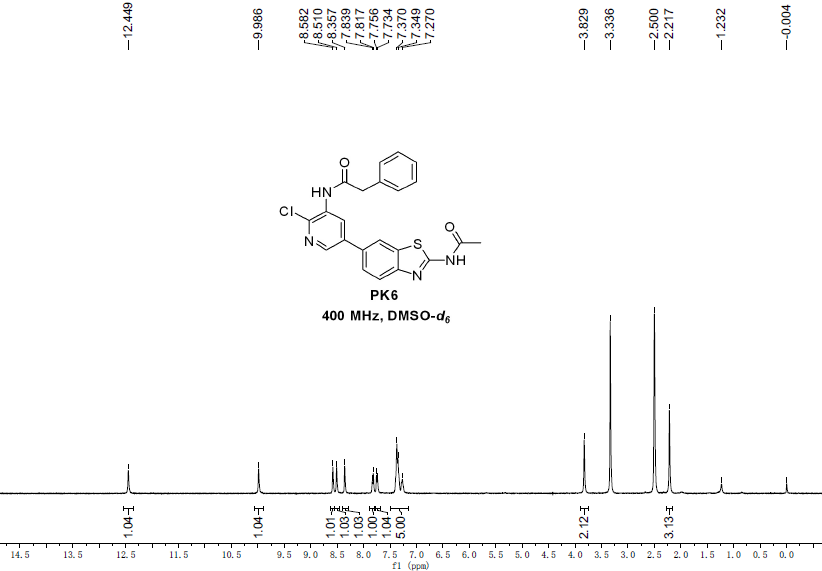

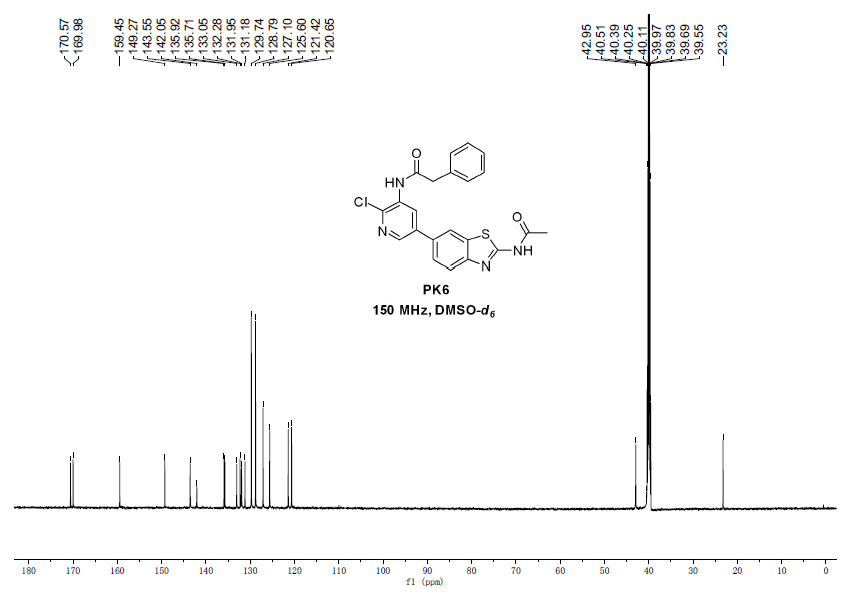


**Supplementary Figure 3**. ^1^H and ^13^C NMR spectra for **PK6**


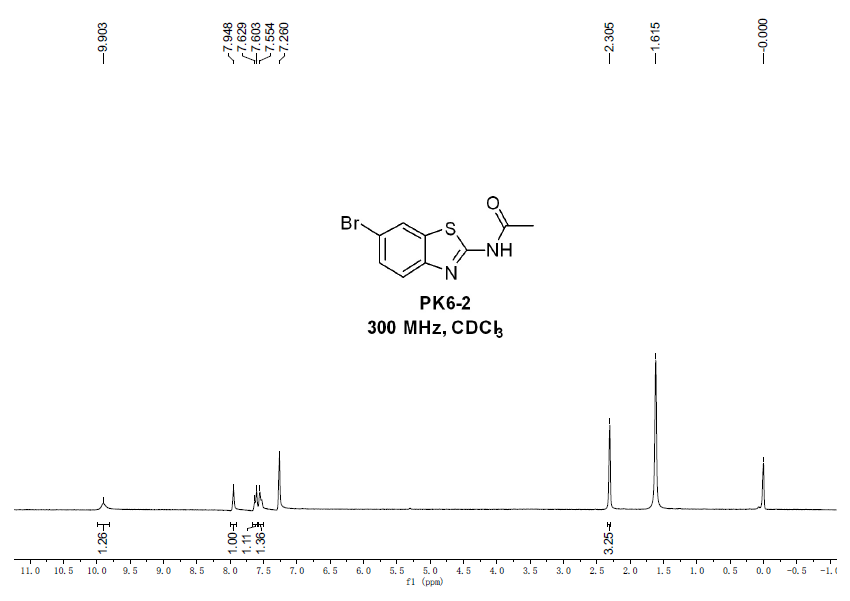


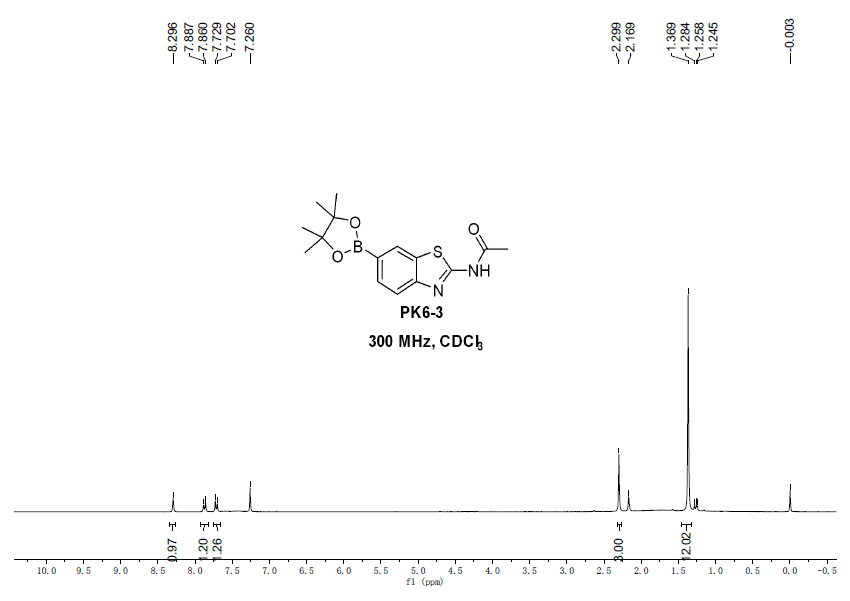


**Supplementary Figure 4**. ^1^H NMR spectra for **PK6-2** and **PK6-3**


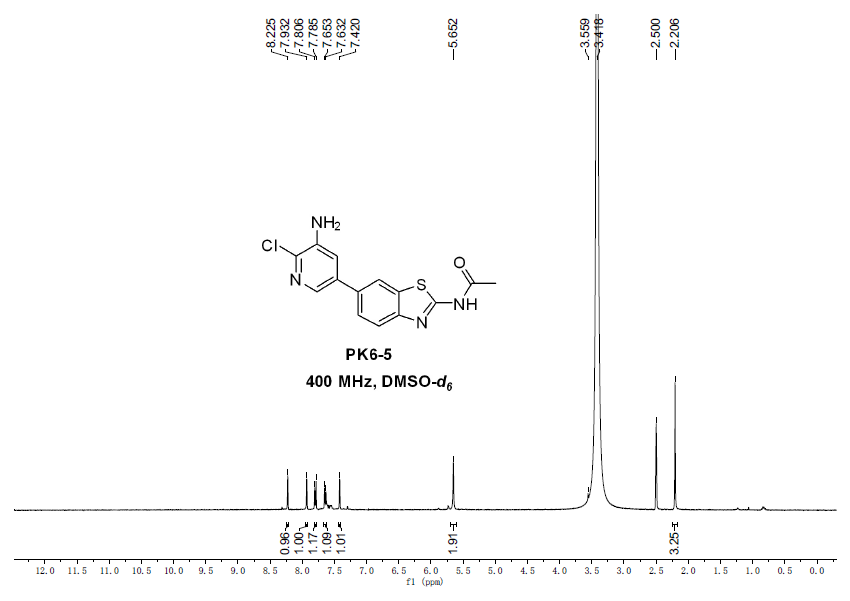


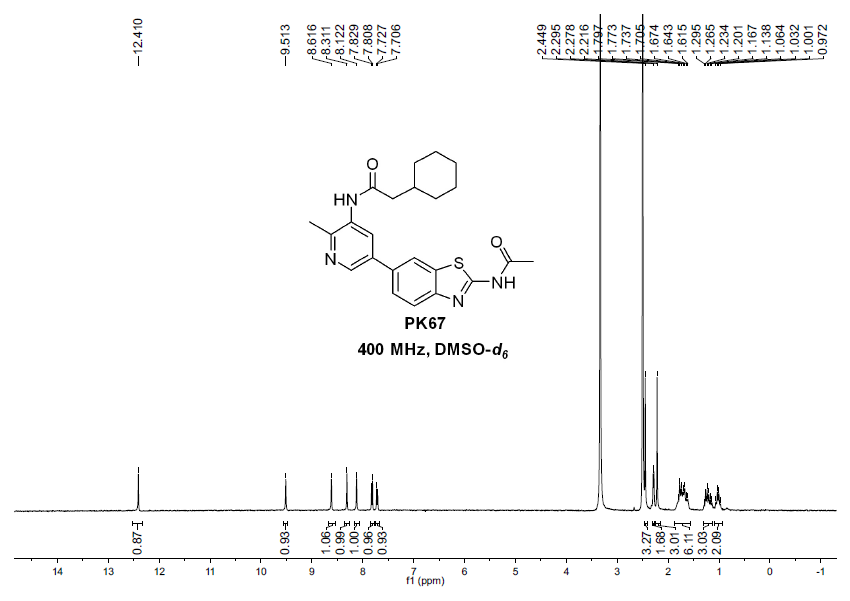


**Supplementary Figure 5**. ^1^H NMR spectra for **PK6-5** and **PK67**


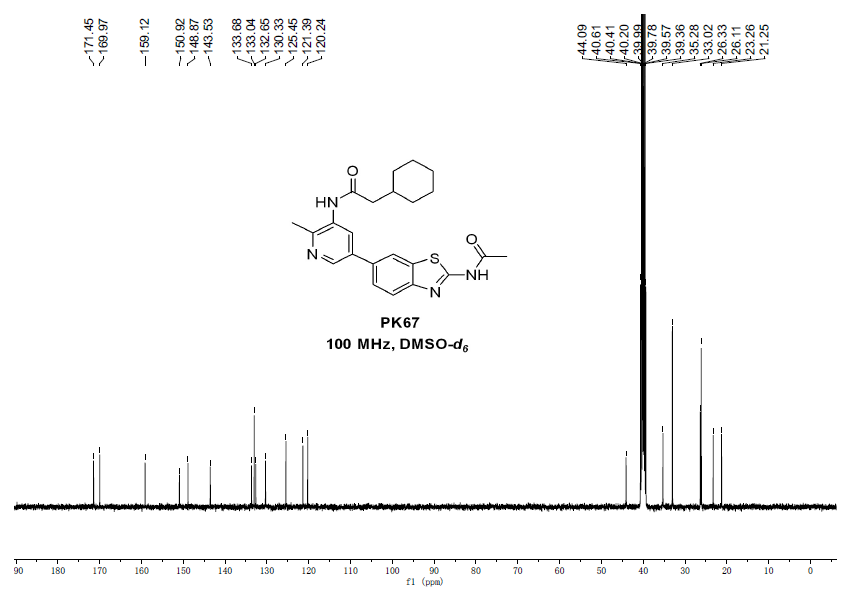


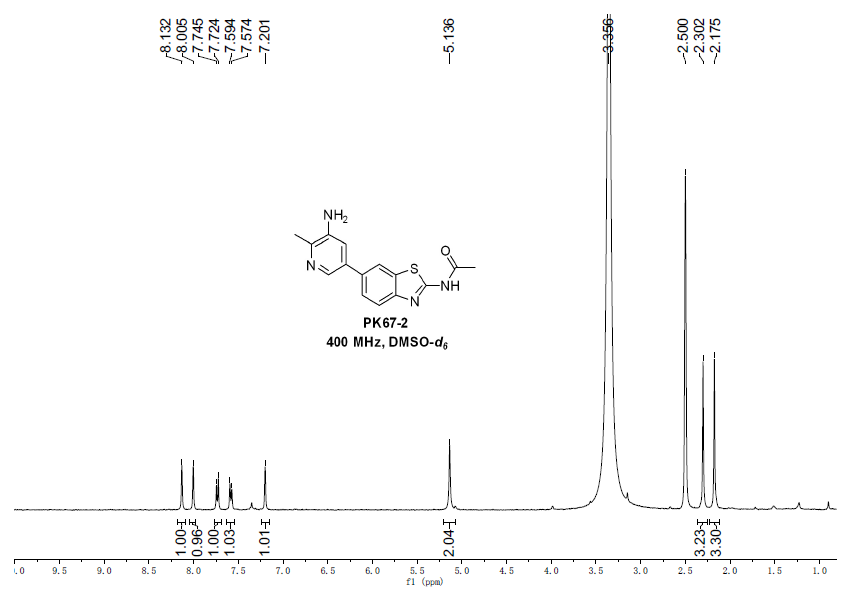


**Supplementary Figure 6**. ^13^C NMR spectra for **PK67** and ^1^H NMR spectra for **PK67-2**


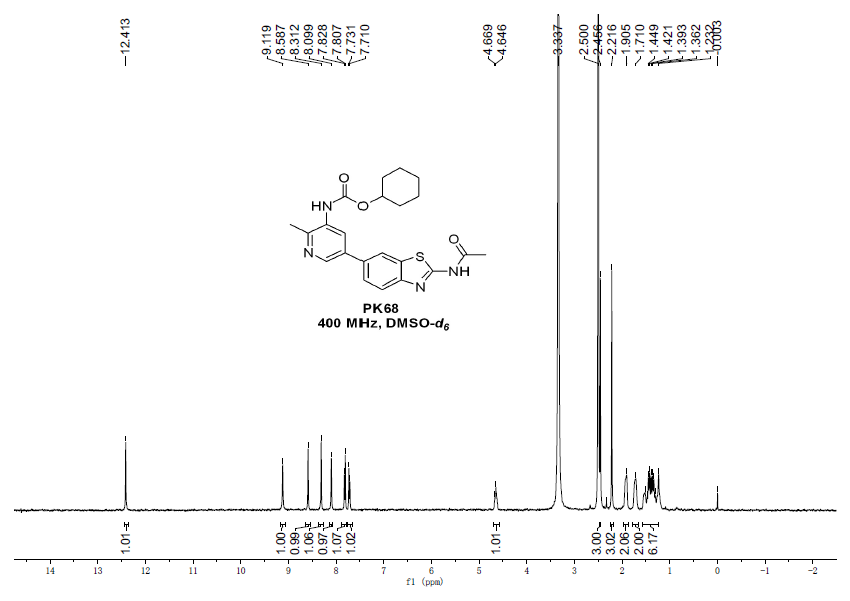

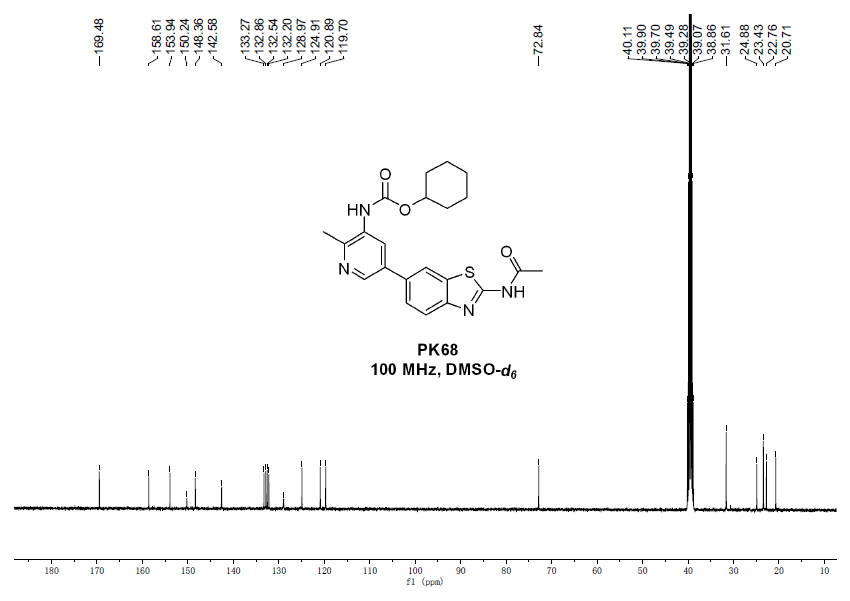


**Supplementary Figure 7**. ^1^H and ^13^C NMR spectra for **PK68**


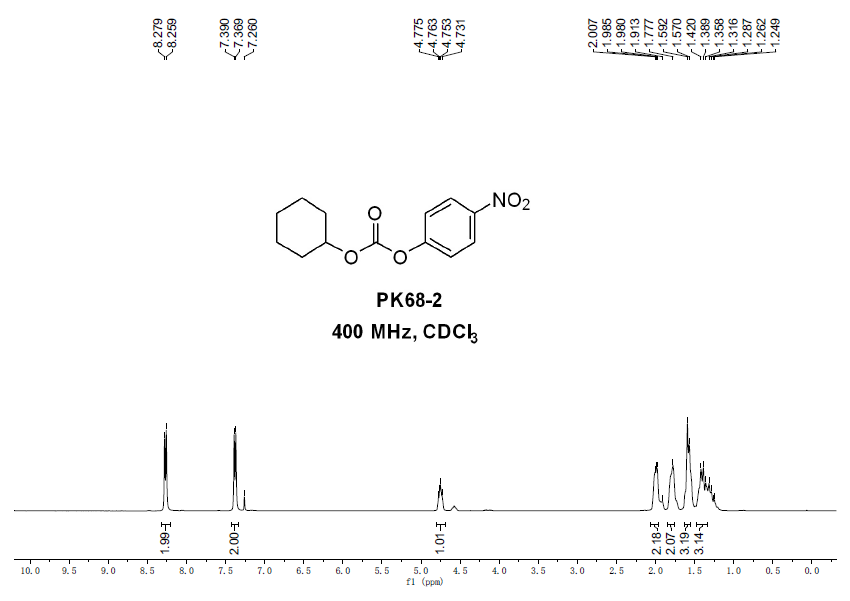


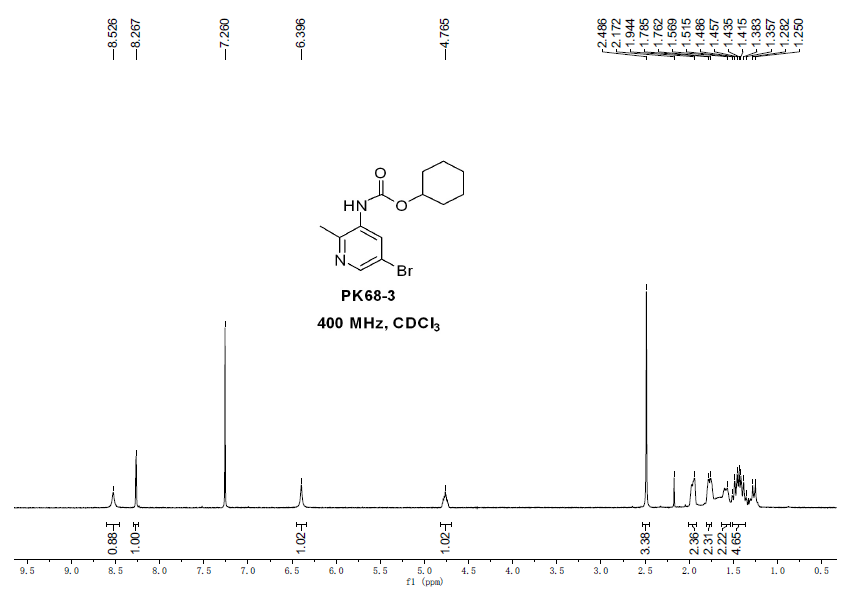


**Supplementary Figure 8**. ^1^H NMR spectra for **PK68-2** and **PK68-3**


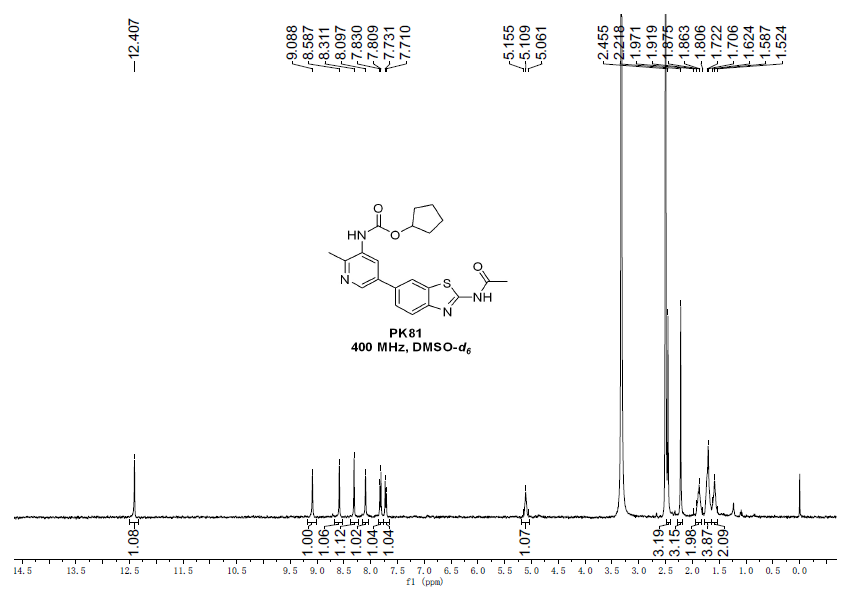


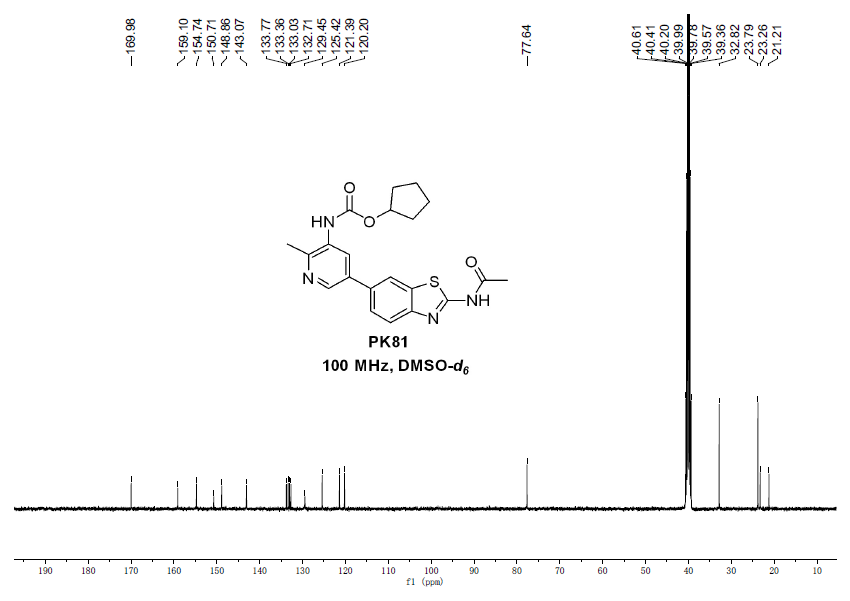


**Supplementary Figure 9**. ^1^H and ^13^C NMR spectra for **PK81**


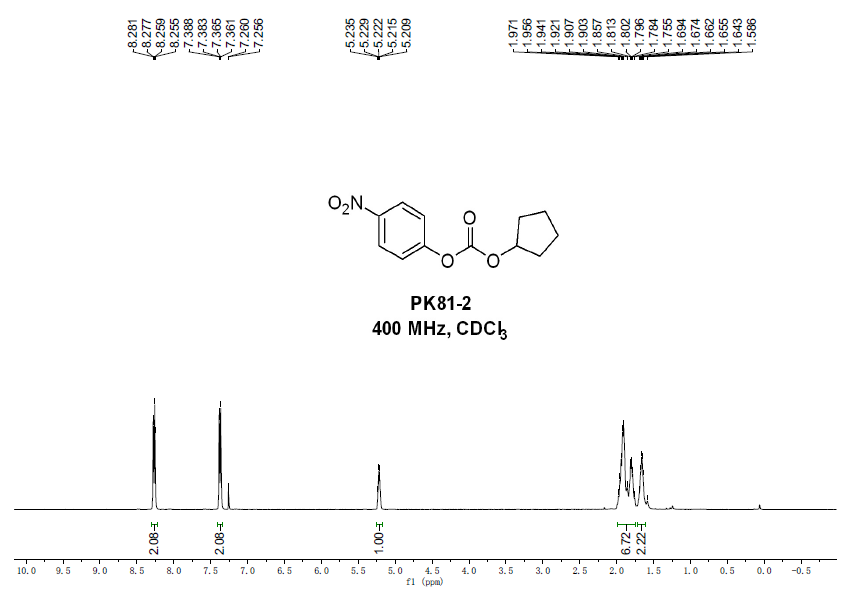


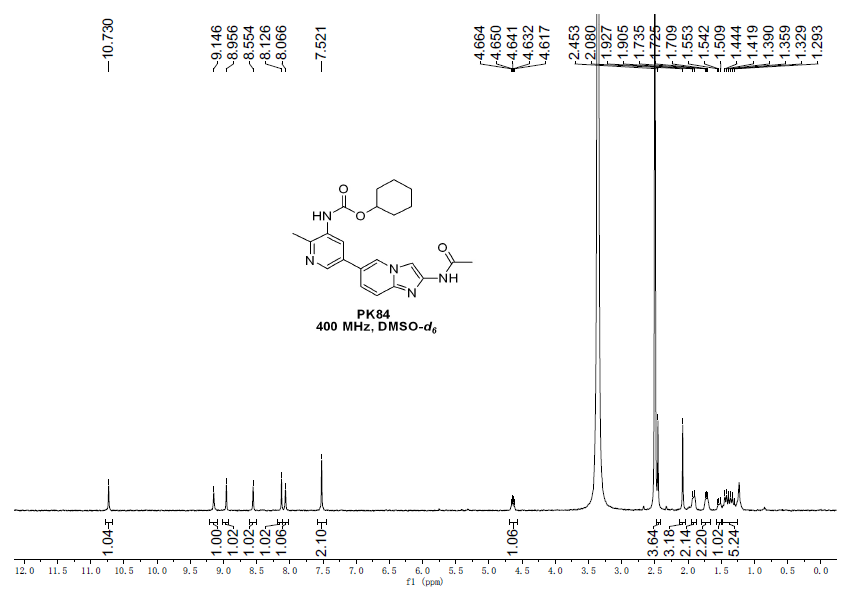


**Supplementary Figure 10**. ^1^H NMR spectra for **PK81-2** and **PK84**


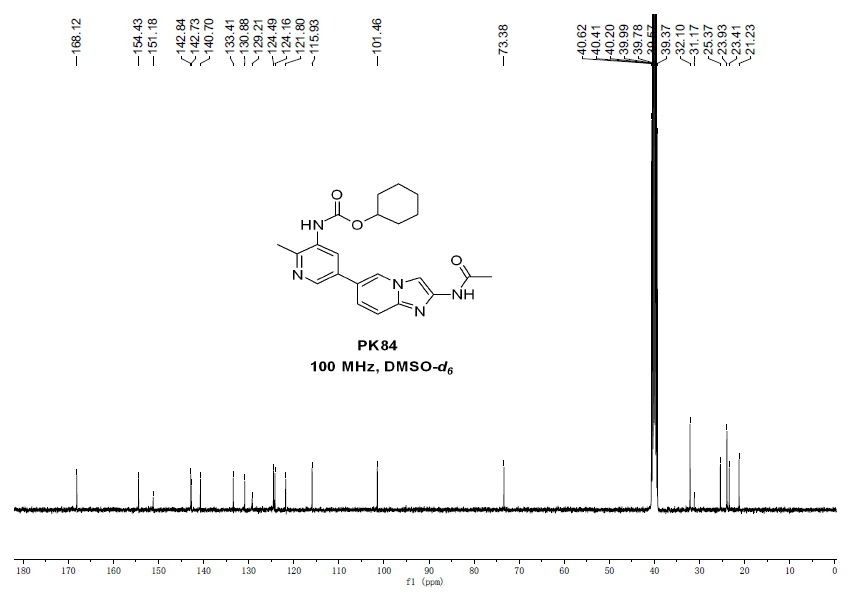


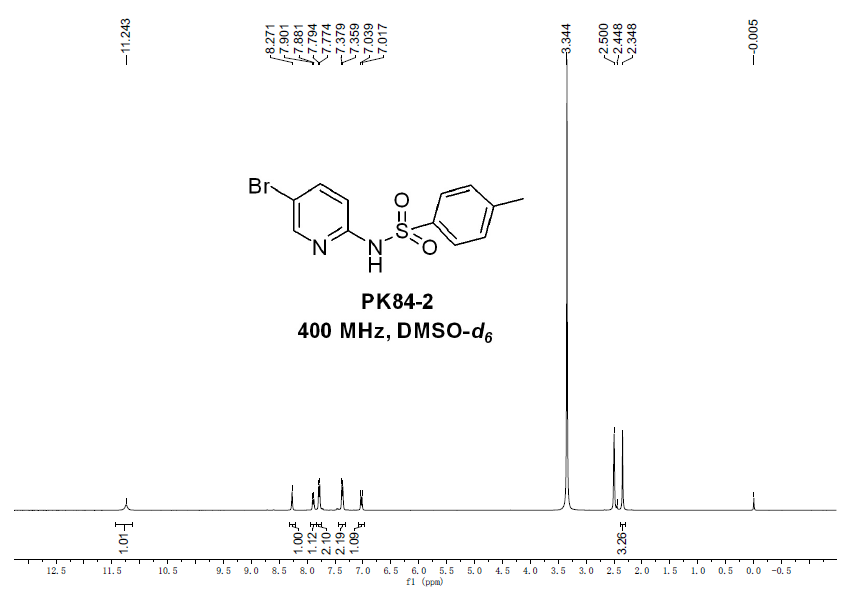


**Supplementary Figure 11**. ^13^C NMR spectra for **PK84** and ^1^H NMR spectra for **PK84-2**


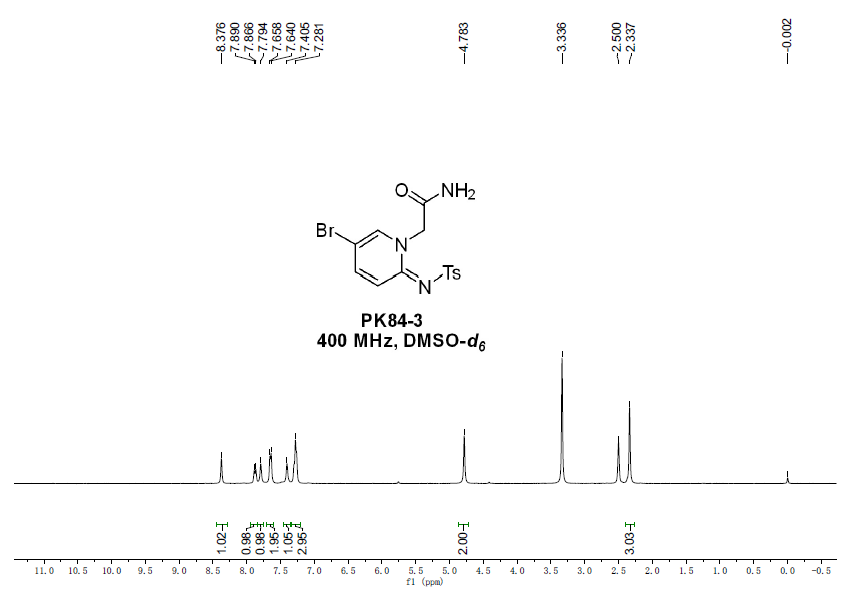

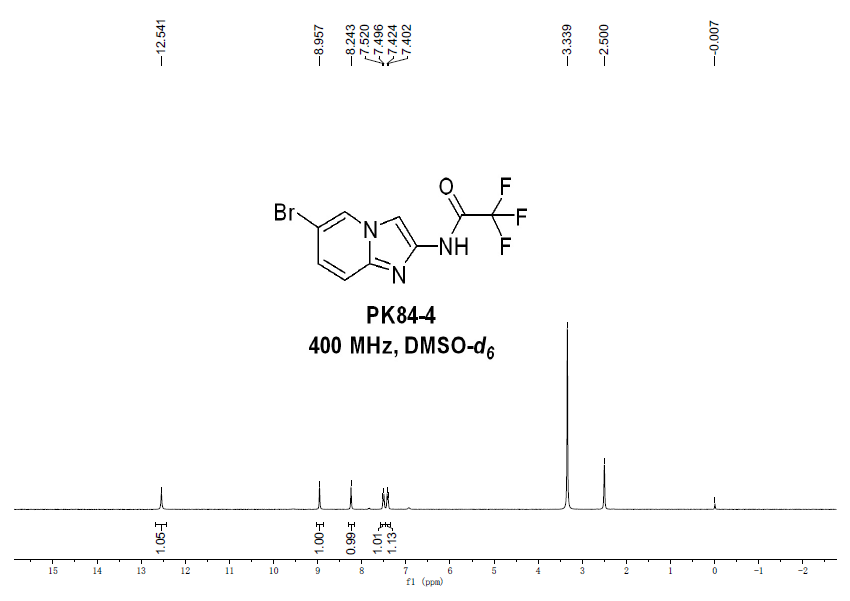


**Supplementary Figure 12**. ^1^H NMR spectra for **PK84-3** and **PK84-4**


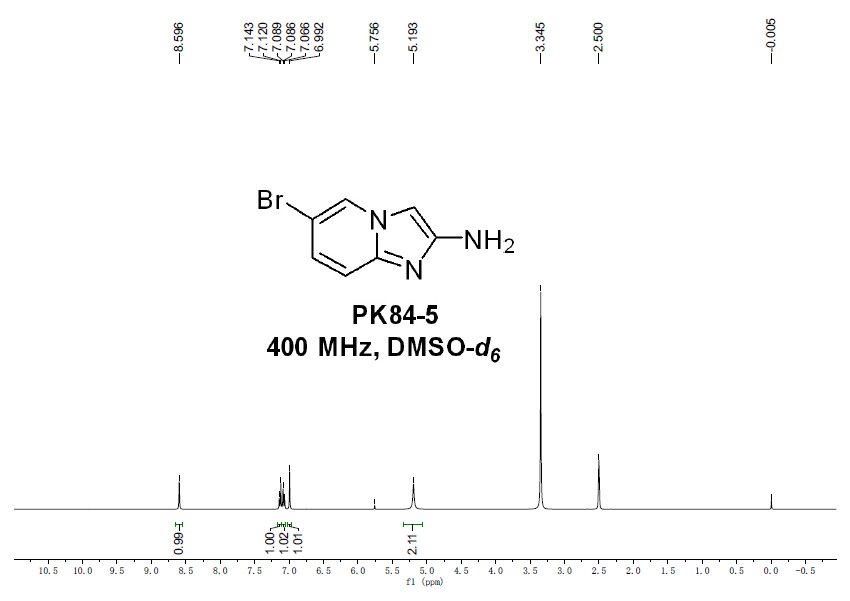


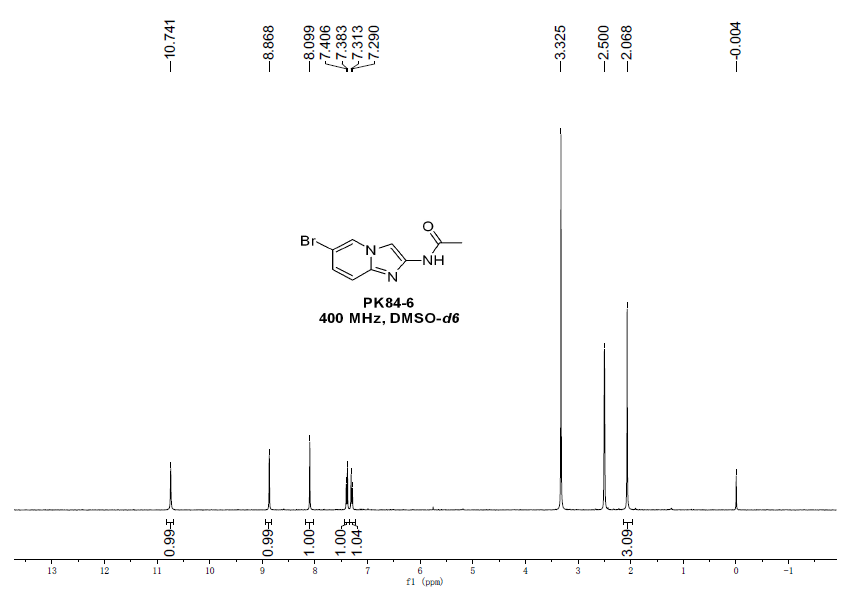


**Supplementary Figure 13**. ^1^H NMR spectra for **PK84-5** and **PK84-6**


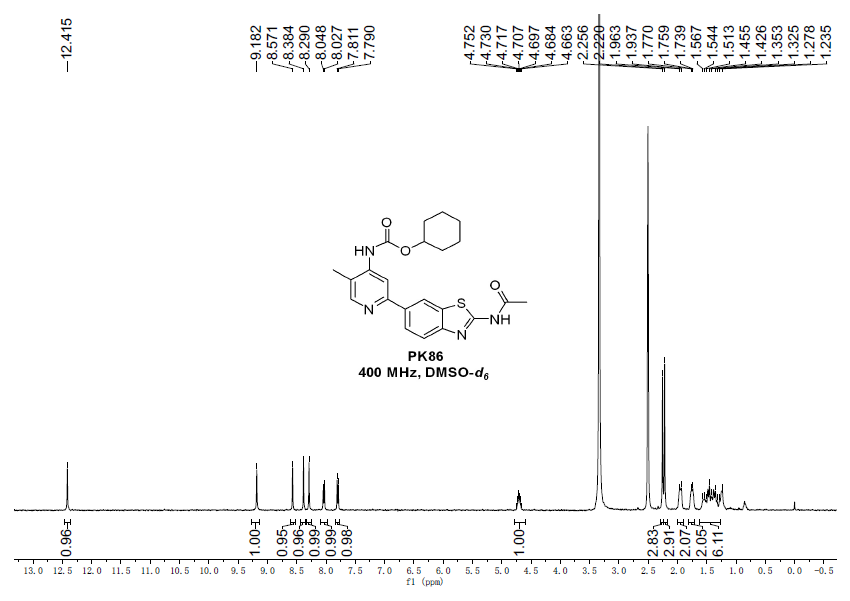


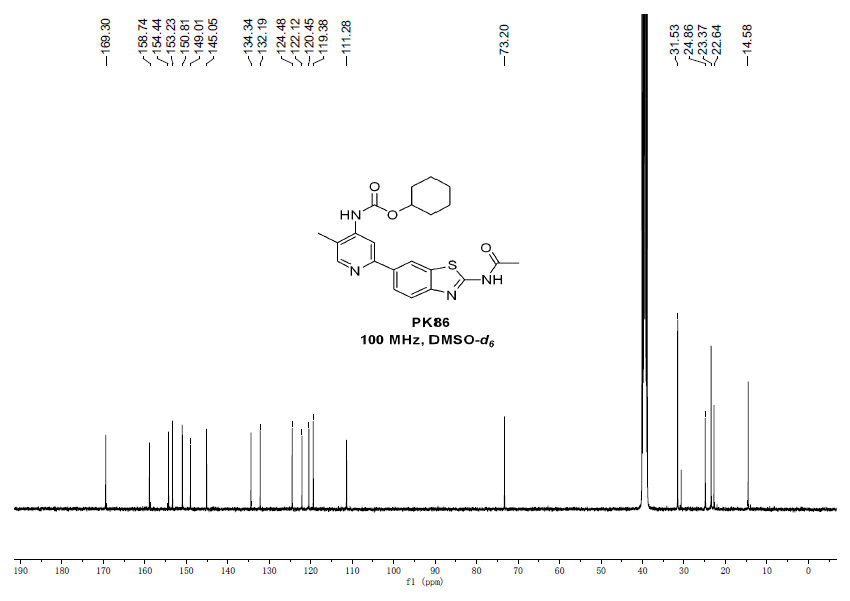


**Supplementary Figure 14**. ^1^H and ^13^C NMR spectra for **PK86**


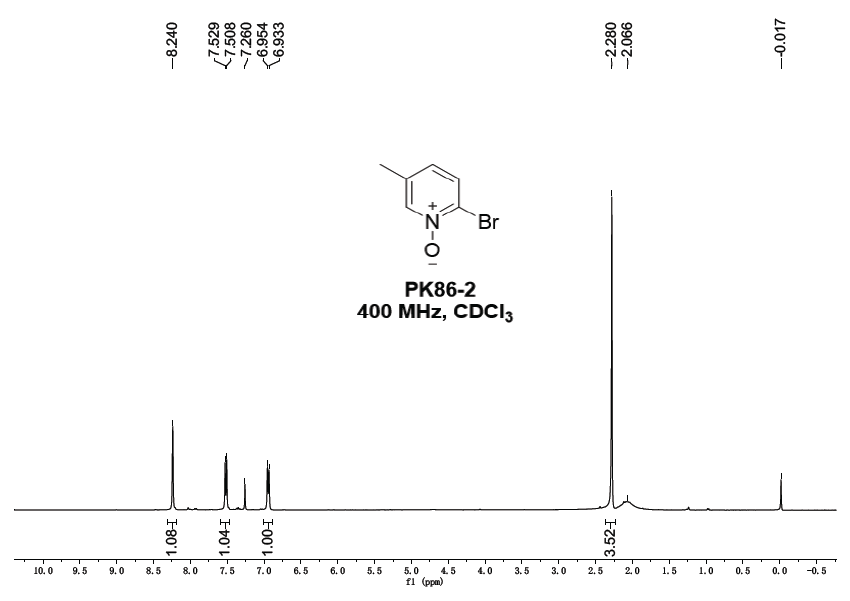


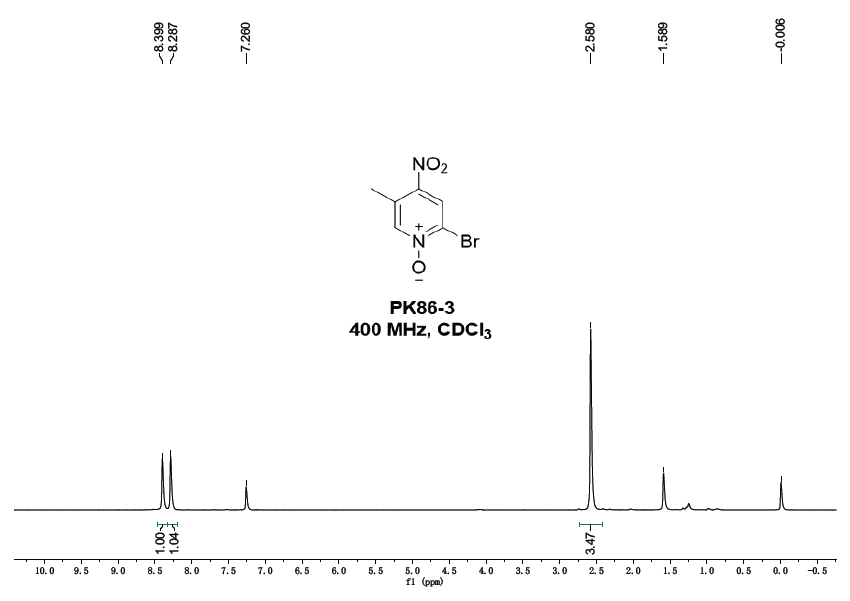


**Supplementary Figure 15**. ^1^H NMR spectra for **PK86-2** and **PK86-3**


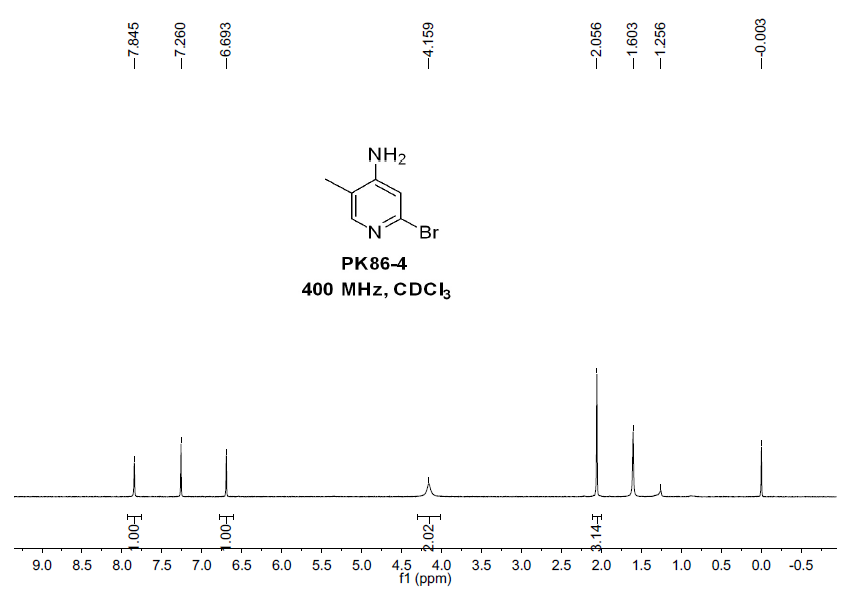


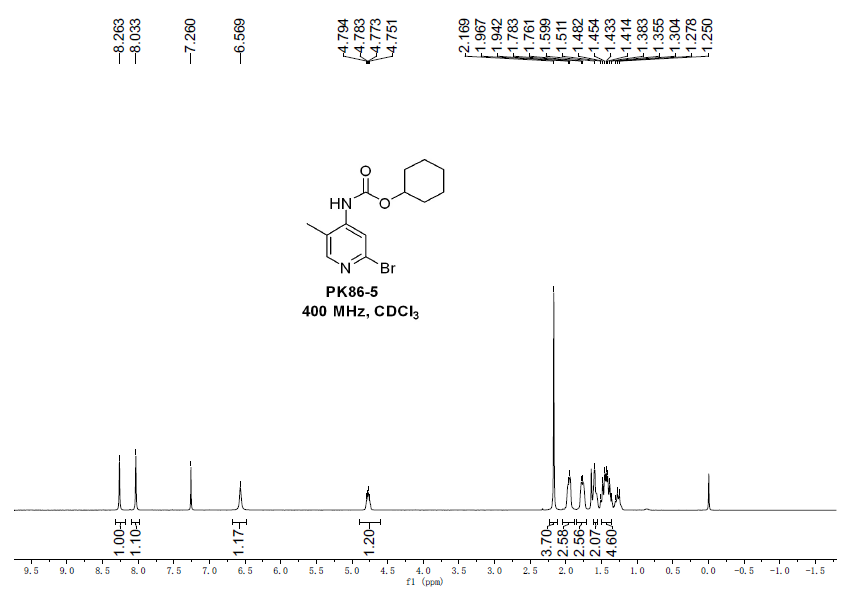


**Supplementary Figure 16**. ^1^H NMR spectra for **PK86-4** and **PK86-5**


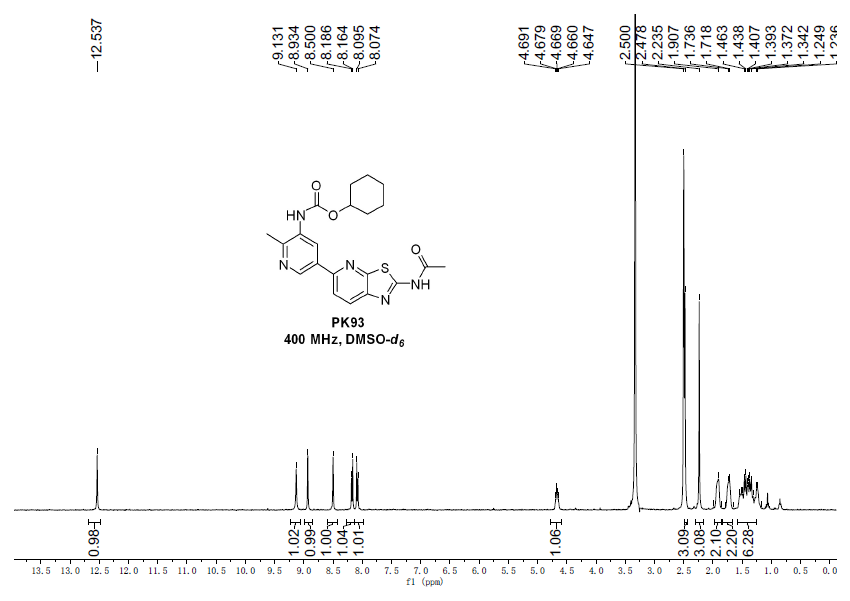


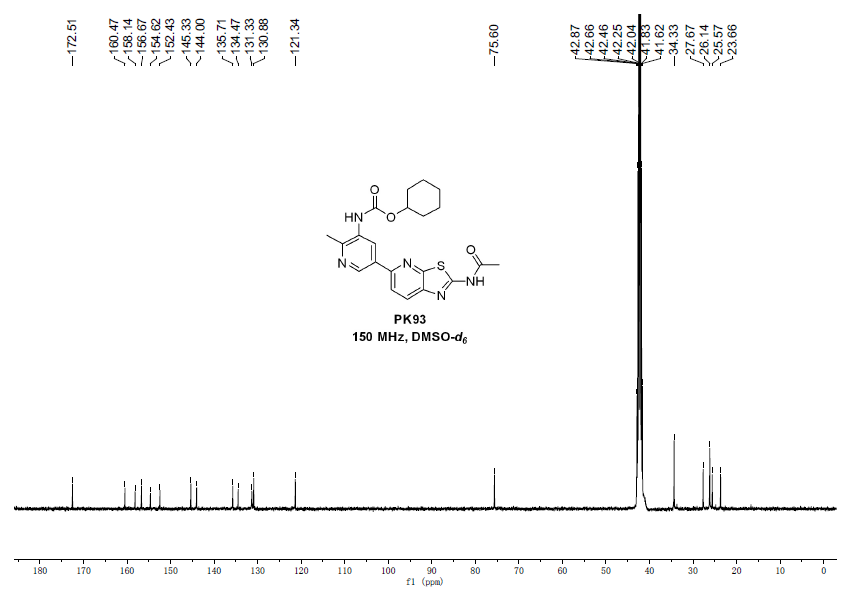


**Supplementary Figure 17**. ^1^H and ^13^C NMR spectra for **PK93**


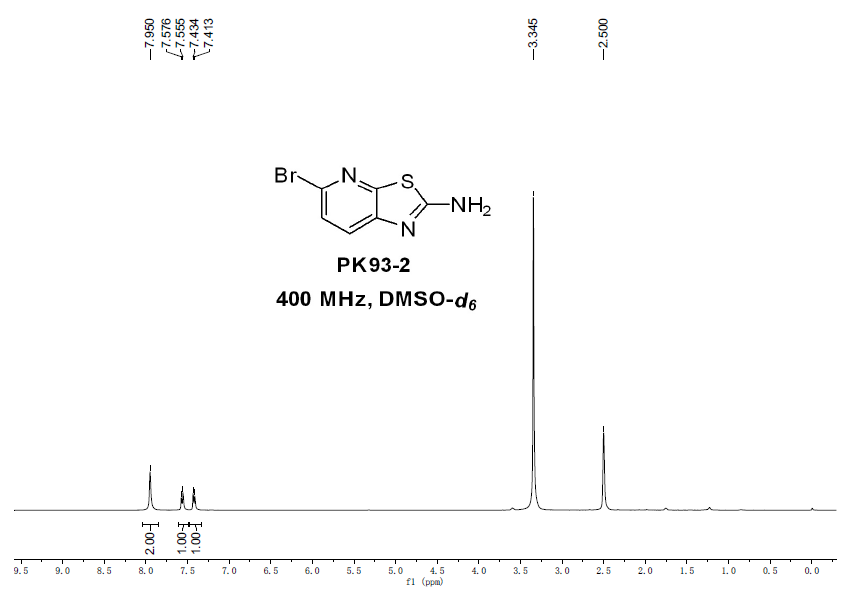

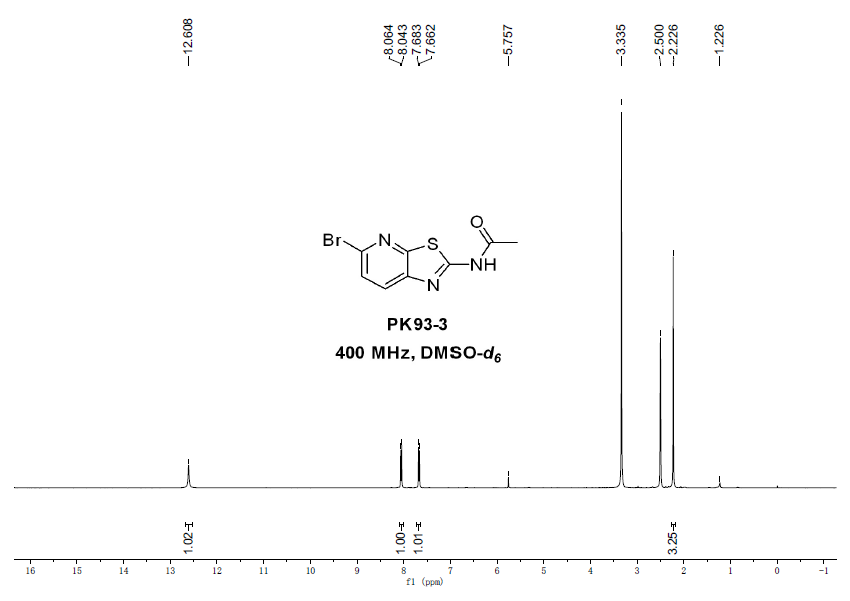


**Supplementary Figure 18**. ^1^H NMR spectra for **PK93-2** and **PK93-3**

**A B**


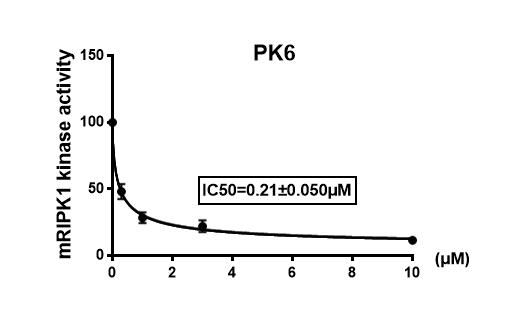

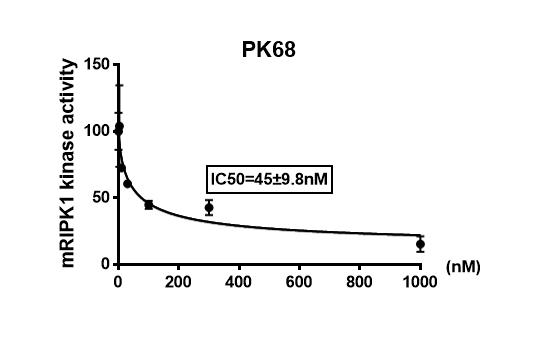


**Supplementary Figure 19. Both PK6 and PK68 were able to block the kinase activity of mouse RIPK1 in vitro.**

In vitro kinase activity assays using recombinant mouse RIPK1 were performed. Recombinant proteins were incubated with PK6(A) and PK68(B) as indicated. Data represent mean value ±standard deviation.


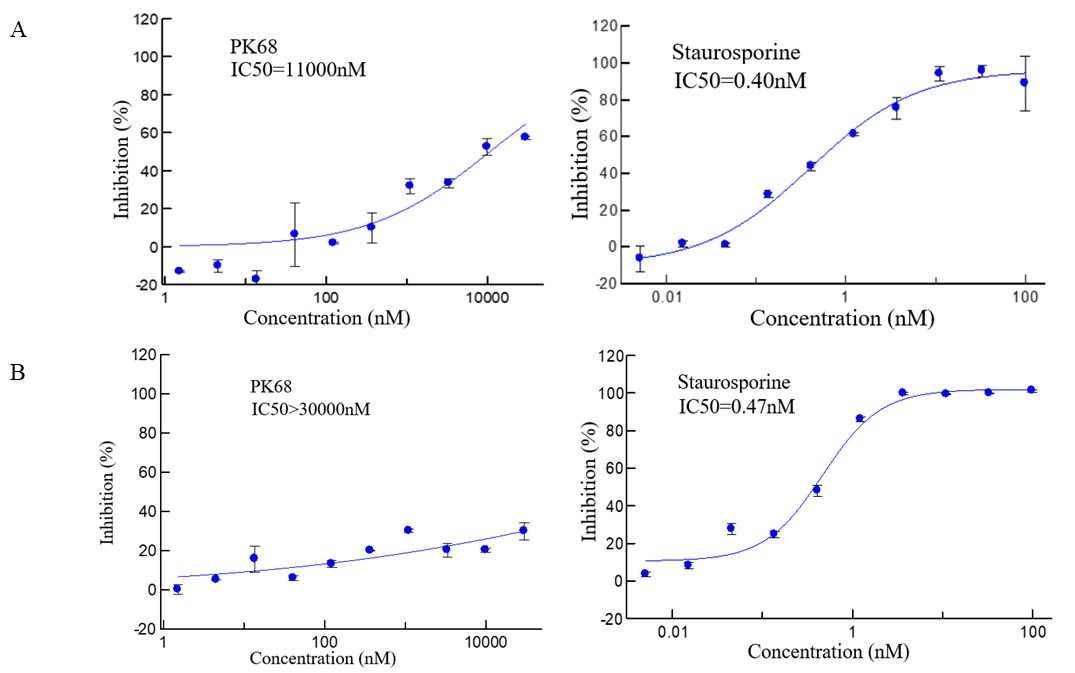


**Supplementary Figure 20. The kinase activity assays of PK68 in TNIK and TRKA in vitro, Staurosporine as control.**

(A) The IC_50_ values were obtained by increasing concentrations of PK68 and Staurosporine in TNIK. (B) The IC_50_ values were obtained by increasing concentrations of PK68 and Staurosporine in TRKA.


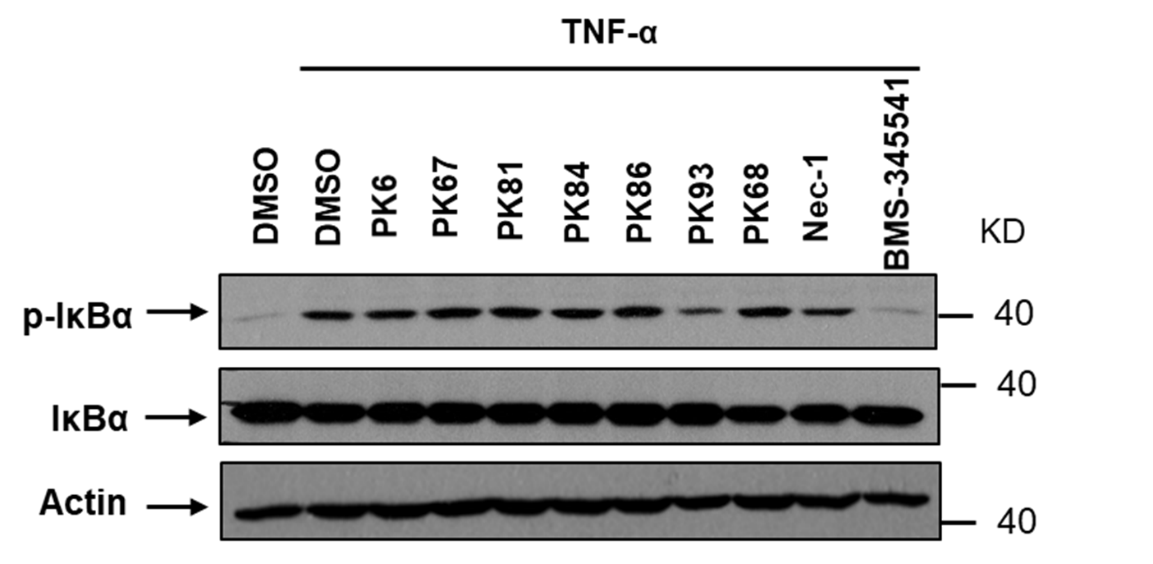


**Supplementary Figure 21. PK6 and PK68 have no obvious effects on NF-κB activation.**

HT-29 cells were pretreated with indicated compounds for 1h prior to stimulation with TNF-α (40ng/ml) for 15 minutes. The levels of IκB-α and phosphor-IκB-α were analyzed by western blot analysis. The NF-κB inhibitor BMS-345541 was used as a positive control.

**A
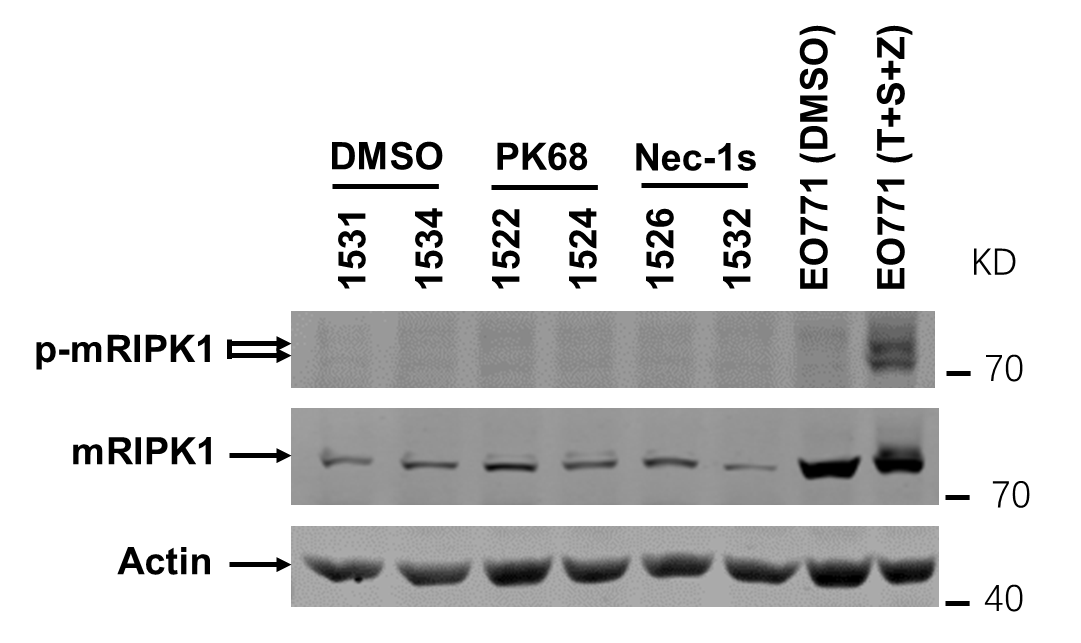
 B**

**
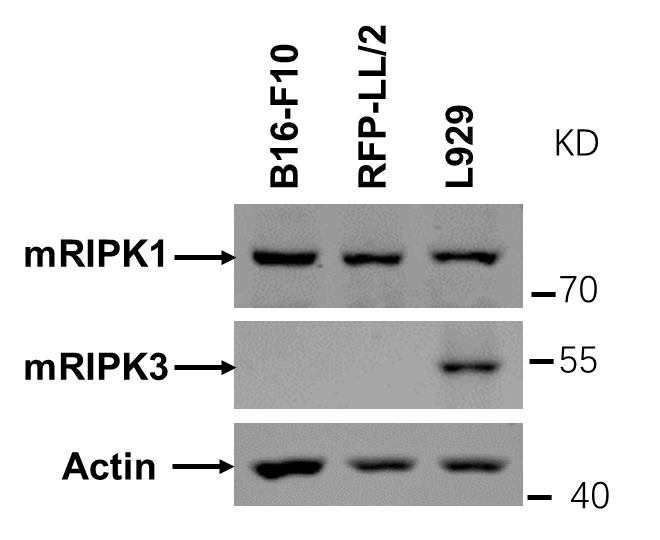
**

**Supplementary Figure 22. Analysis of RIPK1 and p-RIPK1 levels in the tumor cells.**

(A) The expression levels of RIPK1 and RIPK3 in B16-F10 and RFP-LL/2 cells. (B) Two weeks after intravenously injection, RFP^+^ LL/2 cells were isolated from the tumor tissues through flow cytometer sorting for p-mRIPK1 analysis.

**A**


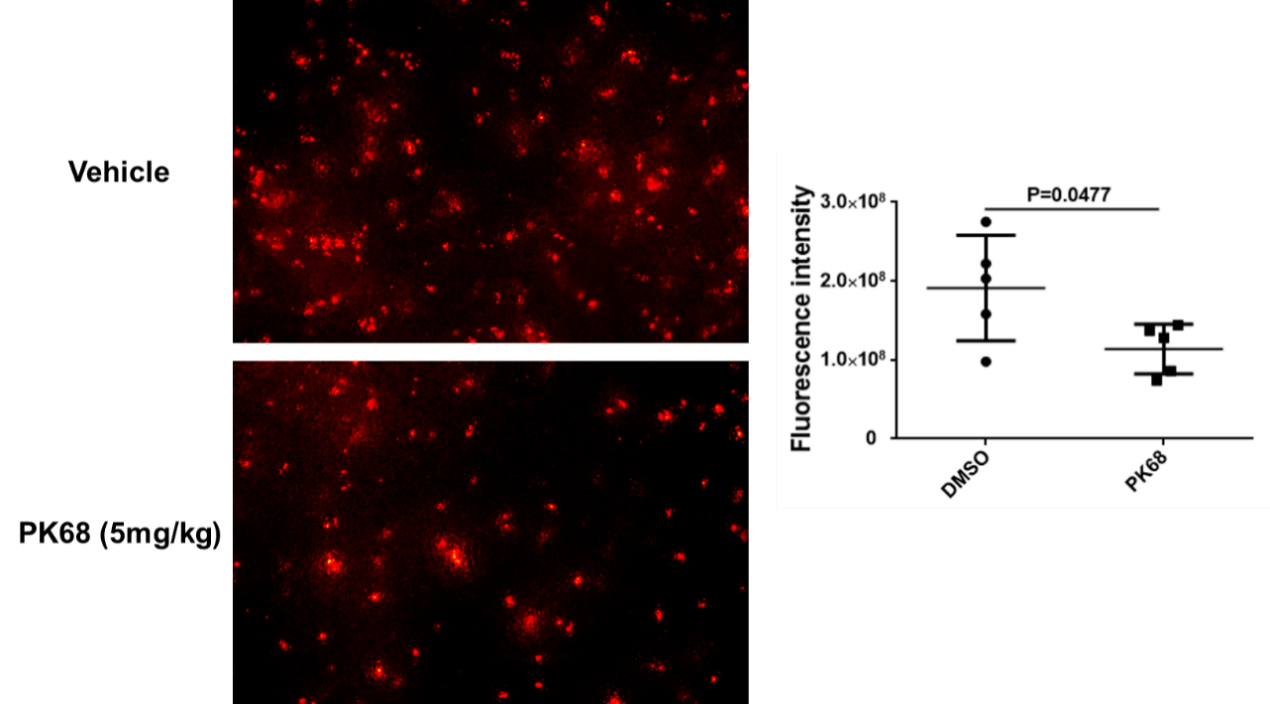


**B**

**Supplementary Figure 23. PK68 attenuates early metastasis of RFP-LL/2 cells.**

Vehicle or PK68 (5mg/kg) was injected intraperitoneally to mice 30min before tumor cells injection, 3h and 6h after tumor cells injection. Six hours after intravenous injection of RFP-LL/2 cells, mice were sacrificed and lung tissues were collected for analysis of RFP-LL/2 metastasis. (A) Representative fluorescent images of RFP-LL/2 metastasis with microscope. RFP-LL/2 metastasis was calculated by RFP fluorescence intensity. (B) The expression level of RFP was analyzed by quantitative real-time PCR.

**
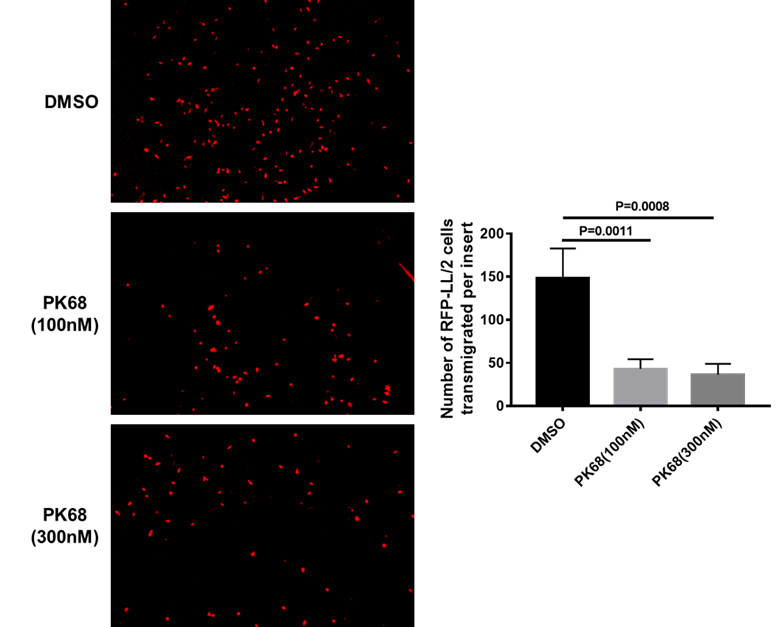
**

**Supplementary Figure 24. PK68 inhibits transmigration of RFP-LL/2 through endothelial cells.**

The primary CD31^+^ endothelial cells isolated from lungs of C57BL/6 mice and RFP-LL/2 cells were used for transendothelial migration assay. Endothelial cells were treated with PK68 for 2 h before addition of RFP-LL/2 tumor cells. Representative images of transmigrated RFP-LL/2 through the endothelial monolayer, and the number of transmigrated RFP-LL/2 cells were analyzed by Image J software.

**A B**

**C**


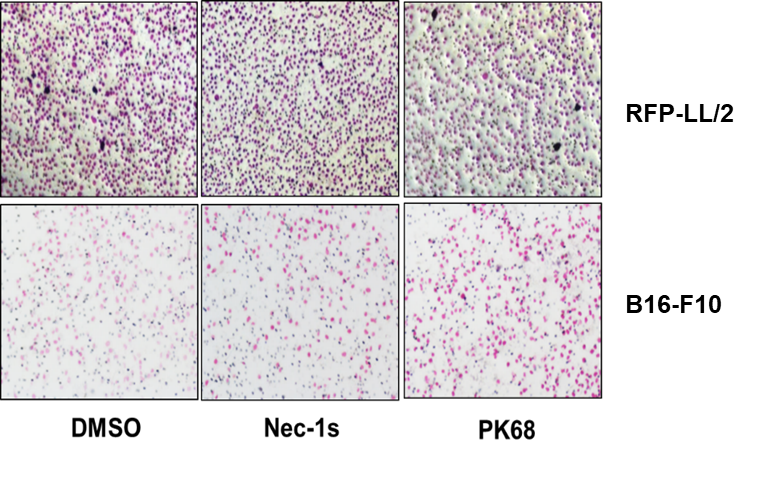


**Supplementary Figure 25. PK68 and Nec-1s have no influence on the proliferation rate and invasion ability of B16-F10 and RFP-LL/2 cells.**

(A-B) B16-F10 and RFP-LL/2 cells were pretreated with indicated compounds for 1h and then cell viability was measured by cell Titer-Glo. (C) B16-F10 and RFP-LL/2 cells were pre-treated with indicated compounds and invasion ability was measured by the transwell assay.

**Table S1. Physicochemical properties of PK6 and PK68.**

| Compound | ClogP^a^ | pKa^a^ | Solubility (μg/mL)^b^ | Melting Point (°C) ^c^ |
| --- | --- | --- | --- | --- |
| PK6 | 3.6 | 1.3 | 0.09 | 293 |
| PK68 | 4.8 | 4.4 | 0.56 | 250 |

^a^ Calculated by ACDlabs. ^b^ Data are measured duplicates in pH= 6.5 FaSSIF using LC/MS/MS. ^c^ Data are determined three times by SMP10 melting point apparatus.

**Table S2. Kinase inhibitory activities of PK68 @1 μM against 369 human proteinKinases^a^**

| Kinase | Activity% | | Kinase | Activity% | |
| --- | --- | --- | --- | --- | --- |
|  | Data1 | Data2 |  | Data1 | Data2 |
| ABL1 | 74.34 | 74.18 | MAPKAPK2 | 101.49 | 100.37 |
| ABL2/ARG | 91.86 | 91.15 | MAPKAPK3 | 102.19 | 100.67 |
| ACK1 | 85.52 | 84.41 | MAPKAPK5/PRAK | 97.04 | 97.02 |
| AKT1 | 99.24 | 99.12 | MARK1 | 97.05 | 96.33 |
| AKT2 | 97.52 | 96.89 | MARK2/PAR-1Ba | 109.69 | 107.76 |
| AKT3 | 99.99 | 98.71 | MARK3 | 104.19 | 103.78 |
| ALK | 96.14 | 94.03 | MARK4 | 102.58 | 101.55 |
| ALK1/ACVRL1 | 95.18 | 91.76 | MEK1 | 99.02 | 97.25 |
| ALK2/ACVR1 | 102.69 | 101.72 | MEK2 | 97.23 | 97.14 |
| ALK3/BMPR1A | 104.90 | 103.05 | MEK3 | 99.68 | 99.61 |
| ALK4/ACVR1B | 95.45 | 95.17 | MEK5 | 97.53 | 97.06 |
| ALK5/TGFBR1 | 95.06 | 94.67 | MEKK1 | 95.34 | 92.88 |
| ALK6/BMPR1B | 92.46 | 91.83 | MEKK2 | 89.82 | 85.76 |
| ARAF | 60.51 | 60.01 | MEKK3 | 114.75 | 111.45 |
| ARK5/NUAK1 | 99.44 | 98.37 | MEKK6 | 91.34 | 88.83 |
| ASK1/MAP3K5 | 95.20 | 93.46 | MELK | 101.96 | 100.40 |
| Aurora A | 95.39 | 95.24 | MINK/MINK1 | 90.20 | 88.92 |
| Aurora B | 83.66 | 83.50 | MKK4 | 99.06 | 98.91 |
| Aurora C | 94.08 | 92.22 | MKK6 | 108.62 | 107.08 |
| AXL | 98.79 | 98.35 | MKK7 | 98.00 | 93.03 |
| BLK | 95.36 | 93.58 | MLCK/MYLK | 99.67 | 97.43 |
| BMPR2 | 95.53 | 95.12 | MLCK2/MYLK2 | 51.31 | 50.92 |
| BMX/ETK | 99.88 | 99.71 | MLK1/MAP3K9 | 99.71 | 99.64 |
| BRAF | 84.75 | 83.61 | MLK2/MAP3K10 | 57.02 | 55.63 |
| BRK | 97.45 | 96.14 | MLK3/MAP3K11 | 82.87 | 82.77 |
| BRSK1 | 102.25 | 101.25 | MLK4 | 106.52 | 104.45 |
| BRSK2 | 100.05 | 98.88 | MNK1 | 100.17 | 99.13 |
| BTK | 91.15 | 90.38 | MNK2 | 90.48 | 88.77 |
| c-Kit | 77.47 | 75.47 | MRCKa/CDC42BPA | 103.92 | 101.41 |
| c-MER | 86.04 | 84.20 | MRCKb/CDC42BPB | 103.93 | 101.65 |
| c-MET | 102.47 | 101.99 | MSK1/RPS6KA5 | 93.99 | 93.86 |
| c-Src | 96.26 | 95.17 | MSK2/RPS6KA4 | 106.63 | 106.42 |
| CAMK1a | 98.59 | 98.23 | MSSK1/STK23 | 103.23 | 100.79 |
| CAMK1b | 101.08 | 99.49 | MST1/STK4 | 100.44 | 100.43 |
| CAMK1d | 102.99 | 100.76 | MST2/STK3 | 109.32 | 109.16 |
| CAMK1g | 92.98 | 92.94 | MST3/STK24 | 102.21 | 100.31 |
| CAMK2a | 100.48 | 99.17 | MST4 | 102.15 | 101.18 |
| CAMK2b | 101.68 | 99.42 | MUSK | 75.86 | 70.88 |
| CAMK2d | 98.90 | 98.13 | MYLK3 | 100.75 | 100.10 |
| CAMK2g | 107.23 | 105.45 | MYLK4 | 92.70 | 89.75 |
| CAMK4 | 84.85 | 84.49 | MYO3A | 92.22 | 91.22 |
| CAMKK1 | 112.70 | 111.65 | MYO3b | 99.92 | 99.36 |
| CAMKK2 | 80.12 | 79.89 | NEK1 | 96.01 | 95.75 |
| CDC7/DBF4 | 109.56 | 104.41 | NEK11 | 85.41 | 81.13 |
| CDK1/cyclin A | 97.23 | 96.92 | NEK2 | 114.62 | 112.29 |
| CDK1/cyclin B | 97.60 | 97.32 | NEK3 | 119.35 | 116.50 |
| CDK1/cyclin E | 101.94 | 101.92 | NEK4 | 98.92 | 97.97 |
| CDK14/cyclin Y (PFTK1) | 97.41 | 96.97 | NEK5 | 95.51 | 95.10 |
| CDK16/cyclin Y (PCTAIRE) | 99.16 | 96.14 | NEK6 | 98.39 | 93.63 |
| CDK17/cyclin Y (PCTK2) | 78.99 | 77.31 | NEK7 | 104.85 | 103.37 |
| CDK18/cyclin Y (PCTK3) | 107.23 | 106.96 | NEK8 | 79.93 | 79.27 |
| CDK19/cyclin C | 100.89 | 99.34 | NEK9 | 90.87 | 90.50 |
| CDK2/cyclin A | 98.51 | 97.94 | NIM1 | 97.52 | 96.79 |
| CDK2/Cyclin A1 | 98.52 | 96.71 | NLK | 102.67 | 98.89 |
| CDK2/cyclin E | 93.46 | 91.99 | OSR1/OXSR1 | 104.67 | 102.40 |
| CDK2/cyclin O | 99.83 | 98.77 | P38a/MAPK14 | 106.40 | 102.59 |
| CDK3/cyclin E | 91.72 | 91.48 | P38b/MAPK11 | 103.21 | 102.60 |
| CDK4/cyclin D1 | 88.84 | 88.58 | P38d/MAPK13 | 95.84 | 95.45 |
| CDK4/cyclin D3 | 94.25 | 93.75 | P38g | 109.23 | 105.38 |
| CDK5/p25 | 94.11 | 92.68 | p70S6K/RPS6KB1 | 101.87 | 101.85 |
| CDK5/p35 | 102.00 | 100.63 | p70S6Kb/RPS6KB2 | 98.77 | 95.50 |
| CDK6/cyclin D1 | 93.77 | 93.73 | PAK1 | 101.64 | 101.03 |
| CDK6/cyclin D3 | 82.56 | 81.98 | PAK2 | 105.05 | 104.24 |
| CDK7/cyclin H | 89.20 | 87.73 | PAK3 | 102.74 | 99.63 |
| CDK9/cyclin K | 105.52 | 104.96 | PAK4 | 103.86 | 101.97 |
| CDK9/cyclin T1 | 111.26 | 110.56 | PAK5 | 101.66 | 98.16 |
| CDK9/cyclin T2 | 90.71 | 88.23 | PAK6 | 93.60 | 92.62 |
| CHK1 | 87.26 | 87.21 | PASK | 93.04 | 91.47 |
| CHK2 | 99.06 | 98.81 | PBK/TOPK | 98.40 | 97.84 |
| CK1a1 | 96.96 | 94.49 | PDGFRa | 84.12 | 83.70 |
| CK1a1L | 106.01 | 103.24 | PDGFRb | 76.67 | 75.13 |
| CK1d | 105.49 | 103.42 | PDK1/PDPK1 | 92.72 | 90.43 |
| CK1epsilon | 78.19 | 74.35 | PEAK1 | 103.36 | 100.13 |
| CK1g1 | 90.68 | 89.50 | PHKg1 | 93.62 | 93.46 |
| CK1g2 | 97.75 | 96.19 | PHKg2 | 99.97 | 98.74 |
| CK1g3 | 87.13 | 87.02 | PIM1 | 101.98 | 100.16 |
| CK2a | 95.18 | 90.49 | PIM2 | 113.58 | 111.50 |
| CK2a2 | 100.69 | 100.37 | PIM3 | 104.37 | 101.62 |
| CLK1 | 81.88 | 80.72 | PKA | 105.47 | 105.35 |
| CLK2 | 80.49 | 77.67 | PKAcb | 102.56 | 100.88 |
| CLK3 | 100.92 | 99.85 | PKAcg | 95.60 | 94.66 |
| CLK4 | 71.04 | 70.07 | PKCa | 99.45 | 98.94 |
| COT1/MAP3K8 | 95.22 | 94.93 | PKCb1 | 84.84 | 84.55 |
| CSK | 92.76 | 91.36 | PKCb2 | 100.07 | 98.56 |
| CTK/MATK | 93.82 | 93.51 | PKCd | 100.89 | 100.58 |
| DAPK1 | 88.50 | 87.16 | PKCepsilon | 99.50 | 98.93 |
| DAPK2 | 109.46 | 107.36 | PKCeta | 92.77 | 90.40 |
| DCAMKL1 | 87.70 | 87.59 | PKCg | 95.83 | 95.02 |
| DCAMKL2 | 100.33 | 99.58 | PKCiota | 97.00 | 92.87 |
| DDR1 | 81.72 | 80.91 | PKCmu/PRKD1 | 96.53 | 95.71 |
| DDR2 | 104.85 | 103.50 | PKCnu/PRKD3 | 102.10 | 101.06 |
| DLK/MAP3K12 | 103.07 | 94.64 | PKCtheta | 101.10 | 97.50 |
| DMPK | 101.38 | 100.30 | PKCzeta | 103.13 | 102.04 |
| DMPK2 | 98.27 | 96.42 | PKD2/PRKD2 | 93.89 | 90.63 |
| DRAK1/STK17A | 91.27 | 90.72 | PKG1a | 90.28 | 90.22 |
| DYRK1/DYRK1A | 97.41 | 95.93 | PKG1b | 85.01 | 84.59 |
| DYRK1B | 99.44 | 97.55 | PKG2/PRKG2 | 107.53 | 106.03 |
| DYRK2 | 86.26 | 85.35 | PKN1/PRK1 | 97.85 | 96.02 |
| DYRK3 | 82.95 | 81.73 | PKN2/PRK2 | 62.61 | 62.44 |
| DYRK4 | 102.37 | 102.24 | PKN3/PRK3 | 94.14 | 92.58 |
| EGFR | 99.65 | 99.30 | PLK1 | 94.58 | 93.68 |
| EPHA1 | 100.87 | 100.48 | PLK2 | 102.29 | 102.02 |
| EPHA2 | 95.23 | 94.92 | PLK3 | 104.65 | 104.01 |
| EPHA3 | 101.51 | 98.26 | PLK4/SAK | 91.80 | 89.27 |
| EPHA4 | 92.81 | 92.78 | PRKX | 104.46 | 103.46 |
| EPHA5 | 101.70 | 100.43 | PYK2 | 95.90 | 95.64 |
| EPHA6 | 98.07 | 97.58 | RAF1 | 76.08 | 75.37 |
| EPHA7 | 86.89 | 85.80 | RET | 67.68 | 66.89 |
| EPHA8 | 100.43 | 99.05 | RIPK2 | 91.23 | 85.19 |
| EPHB1 | 95.46 | 95.01 | RIPK3 | 105.97 | 104.99 |
| EPHB2 | 102.27 | 101.30 | RIPK4 | 94.53 | 92.60 |
| EPHB3 | 98.76 | 98.23 | RIPK5 | 102.79 | 102.45 |
| EPHB4 | 101.04 | 99.18 | ROCK1 | 101.46 | 99.31 |
| ERBB2/HER2 | 99.10 | 97.32 | ROCK2 | 95.51 | 95.49 |
| ERBB4/HER4 | 99.70 | 99.02 | RON/MST1R | 99.59 | 99.01 |
| ERK1 | 91.60 | 87.90 | ROS/ROS1 | 88.89 | 88.56 |
| ERK2/MAPK1 | 96.88 | 92.60 | RSK1 | 99.95 | 98.15 |
| ERK5/MAPK7 | 99.27 | 98.25 | RSK2 | 92.33 | 90.87 |
| ERK7/MAPK15 | 99.26 | 96.80 | RSK3 | 97.63 | 96.49 |
| ERN1/IRE1 | 88.67 | 88.33 | RSK4 | 104.83 | 103.68 |
| ERN2/IRE2 | 93.12 | 92.56 | SBK1 | 104.09 | 102.75 |
| FAK/PTK2 | 97.96 | 96.95 | SGK1 | 93.79 | 93.47 |
| FER | 101.91 | 100.70 | SGK2 | 100.95 | 99.97 |
| FES/FPS | 96.94 | 96.80 | SGK3/SGKL | 101.79 | 99.59 |
| FGFR1 | 105.39 | 104.83 | SIK1 | 98.41 | 97.74 |
| FGFR2 | 91.01 | 88.90 | SIK2 | 95.49 | 94.58 |
| FGFR3 | 90.51 | 90.12 | SIK3 | 99.36 | 94.84 |
| FGFR4 | 89.91 | 89.46 | SLK/STK2 | 91.88 | 91.29 |
| FGR | 87.23 | 82.32 | SNARK/NUAK2 | 98.74 | 97.10 |
| FLT1/VEGFR1 | 100.05 | 100.00 | SNRK | 94.93 | 90.23 |
| FLT3 | 57.95 | 57.70 | SRMS | 99.71 | 97.95 |
| FLT4/VEGFR3 | 90.95 | 90.37 | SRPK1 | 100.12 | 99.67 |
| FMS | 71.09 | 71.06 | SRPK2 | 101.03 | 100.99 |
| FRK/PTK5 | 101.16 | 97.78 | SSTK/TSSK6 | 91.26 | 91.18 |
| FYN | 104.12 | 102.88 | STK16 | 88.23 | 85.85 |
| GCK/MAP4K2 | 97.60 | 96.82 | STK21/CIT | 124.53 | 121.90 |
| GLK/MAP4K3 | 104.37 | 102.14 | STK22D/TSSK1 | 104.28 | 103.75 |
| GRK1 | 100.22 | 99.42 | STK25/YSK1 | 110.15 | 104.63 |
| GRK2 | 100.51 | 99.02 | STK32B/YANK2 | 107.48 | 106.39 |
| GRK3 | 102.84 | 102.09 | STK32C/YANK3 | 100.70 | 100.29 |
| GRK4 | 106.52 | 103.31 | STK33 | 91.60 | 91.58 |
| GRK5 | 104.37 | 103.56 | STK38/NDR1 | 98.90 | 96.05 |
| GRK6 | 102.06 | 99.89 | STK38L/NDR2 | 101.33 | 100.42 |
| GRK7 | 96.49 | 95.94 | STK39/STLK3 | 95.75 | 92.54 |
| GSK3a | 101.81 | 101.33 | SYK | 99.45 | 98.85 |
| GSK3b | 98.10 | 97.24 | TAK1 | 97.53 | 96.74 |
| Haspin | 94.51 | 94.31 | TAOK1 | 79.77 | 79.31 |
| HCK | 102.42 | 100.14 | TAOK2/TAO1 | 82.61 | 81.88 |
| HGK/MAP4K4 | 84.42 | 82.64 | TAOK3/JIK | 94.64 | 93.52 |
| HIPK1 | 87.43 | 86.01 | TBK1 | 96.99 | 96.55 |
| HIPK2 | 110.87 | 110.22 | TEC | 99.05 | 97.93 |
| HIPK3 | 66.41 | 63.99 | TESK1 | 117.98 | 111.93 |
| HIPK4 | 74.59 | 72.16 | TESK2 | 94.26 | 92.21 |
| HPK1/MAP4K1 | 101.18 | 98.32 | TGFBR2 | 115.24 | 114.72 |
| IGF1R | 104.45 | 103.88 | TIE2/TEK | 83.40 | 82.66 |
| IKKa/CHUK | 116.86 | 107.86 | TLK1 | 99.18 | 97.39 |
| IKKb/IKBKB | 109.47 | 107.97 | TLK2 | 98.10 | 96.86 |
| IKKe/IKBKE | 98.21 | 92.74 | TNIK | 39.03 | 38.77 |
| IR | 81.61 | 81.00 | TNK1 | 87.94 | 86.66 |
| IRAK1 | 92.50 | 90.54 | TRKA | 29.14 | 28.42 |
| IRAK4 | 86.86 | 85.85 | TRKB | 41.23 | 41.21 |
| IRR/INSRR | 98.02 | 96.94 | TRKC | 35.36 | 33.96 |
| ITK | 88.58 | 88.47 | TSSK2 | 98.09 | 97.17 |
| JAK1 | 99.46 | 99.20 | TSSK3/STK22C | 103.82 | 103.77 |
| JAK2 | 98.21 | 96.04 | TTBK1 | 109.66 | 107.17 |
| JAK3 | 92.90 | 92.17 | TTBK2 | 98.42 | 98.08 |
| JNK1 | 98.74 | 97.06 | TXK | 100.86 | 99.58 |
| JNK2 | 104.44 | 101.11 | TYK1/LTK | 99.82 | 98.39 |
| JNK3 | 100.76 | 99.15 | TYK2 | 105.70 | 100.62 |
| KDR/VEGFR2 | 90.27 | 88.68 | TYRO3/SKY | 99.62 | 97.95 |
| KHS/MAP4K5 | 76.54 | 75.34 | ULK1 | 104.98 | 100.13 |
| KSR1 | 101.74 | 101.02 | ULK2 | 102.04 | 101.80 |
| KSR2 | 100.28 | 99.64 | ULK3 | 92.93 | 92.16 |
| LATS1 | 102.64 | 101.83 | VRK1 | 104.99 | 104.78 |
| LATS2 | 94.41 | 93.96 | VRK2 | 94.40 | 93.34 |
| LCK | 62.93 | 62.75 | WEE1 | 95.74 | 94.05 |
| LCK2/ICK | 101.34 | 100.77 | WNK1 | 111.92 | 111.38 |
| LIMK1 | 63.44 | 63.37 | WNK2 | 94.64 | 94.21 |
| LIMK2 | 44.03 | 42.64 | WNK3 | 106.70 | 106.32 |
| LKB1 | 103.02 | 102.07 | YES/YES1 | 89.19 | 87.91 |
| LOK/STK10 | 97.88 | 97.50 | YSK4/MAP3K19 | 95.05 | 94.66 |
| LRRK2 | 67.65 | 66.88 | ZAK/MLTK | 68.57 | 66.51 |
| LYN | 95.31 | 92.90 | ZAP70 | 107.09 | 105.87 |
| LYN B | 97.98 | 97.70 | ZIPK/DAPK3 | 100.31 | 98.55 |
| MAK | 97.04 | 96.81 |  |  |  |

^a^ Compound **PK68** was tested at 1 μM in duplicate against 369 kinases using the RBC kinase panel service of Reaction Biology Corporation. All the ATP concentrations used were 10 μM. The 369 kinase panel did not include RIPK1. Full protocol details are available at **http://www.reactionbiology.com**.

**Detailed Synthetic Procedures**

General reaction progress was monitored by analytical thin layer chromatography performed on silica gel HSGF254 pre-coated plates. Organic solutions were dried over anhydrous Na_2_SO_4_, and the solvents were removed under reduced pressure. Final compounds were purified with silica gel 100-200 mesh for column chromatography. ^1^H NMR and ^13^C NMR were obtained on 300 MHz (Varian), 400 MHz (Varian) or 600 MHz (Varian) spectrometers. Chemical shifts were given in ppm using tetramethylsilane as internal standard. Data for ^1^H NMR are reported as follows: chemical shift, multiplicity (s = singlet, d = doublet, t = triplet, q = quartet, m = multiplet, br = broad), coupling constants and integration. Mass spectra were obtained using an Agilent 1100 LC/MSD Trap SL version Mass Spectrometer. HRMS analysis was recorded on an Agilent 6540 UHD Accurate-Mass QTOF LC/MS. Melting points were determined by a SMP10 melting point apparatus.

1. **Synthesis of PK6**

**PK6-2:** *N*-(6-bromobenzo[*d*]thiazol-2-yl)acetamide

To a stirred solution of PK6-1 (2.5 g, 10.7 mmol) and DMAP (1.3 g, 12.8 mmol) in 20 mL dichloromethane at 0 °C, Ac_2_O (1.2 mL, 13.0 mmol) was added dropwise. After carried out at room temperature overnight, the mixture was quenched with saturated aqueous NaHCO_3_ (100 mL). The resulting precipitate was filtered. The cake was washed with water and dried to give a white solid (2.30 g, 79 %). ^1^H NMR(300 MHz, CDCl_3_) δ 9.90 (br s, 1H), 7.94 (s, 1H), 7.61 (d, *J* = 10.4 Hz, 1H), 7.54 (d, *J* = 10.4 Hz, 1H), 2.31 (s, 3H).

**PK6-3:** *N*-(6-(4,4,5,5-tetramethyl-1,3,2-dioxaborolan-2-yl)benzo-[*d*]thiazol-2-yl)acetamide

A 100 mL flask was charged with PK6-2 (2.1 g, 7.8 mmol), bis(pinacolato)diboron (3.0 g, 11.8 mmol), KOAc (3.0 g, 30.6 mmol) and Pd(dppf)Cl_2_ (560 mg, 0.77 mmol) followed by addition of 50 mL DMSO. The equipment was evacuated and refilled with N_2_ three times. The reaction was carried out at 90 °C for 8 h. After cooled to room temperature, the mixture was filtered. The filtrate was diluted with ethyl acetate ( 200 mL). The organic phase was washed with brine (50 mL*3). The organic phase was dried with Na_2_SO_4_ and the solvent was removed by vacuum. The residue was recrystallized in petroleum ether to give a brown solid (2.4 g, 97 %). ^1^H NMR(300 MHz, CDCl_3_) δ 8.30 (s, 1H), 7.87 (d, *J* = 8.0 Hz, 1H), 7.72 (d, *J* = 8.0 Hz, 1H), 2.30 (s, 3H), 1.37 (s, 12H).

**PK6-5:** *N*-(6-(5-amino-6-chloropyridin-3-yl)benzo[*d*]thiazol-2-yl)acetamide

A mixture solution of dioxane/H_2_O (10 mL/0.5 mL) were added 5-bromo-2-chloropyridin-3-amine (200 mg, 0.96 mmol), PK6-3 (255 mg, 0.8 mmol), K_2_CO_3_ (332 mg, 2.41 mmol) and Pd(PPh_3_)_4_ (90 mg, 0.08 mmol). The equipment was evacuated and refilled with N_2_ three times. The reaction was carried out at 80 °C for 8 h. After cooled to room temperature, the solvent was concentrated and the residue was purified by silica gel column chromatography (dichloromethane/methanol = 50/1) to give the title compound as a white solid (246 mg, 96 %). ^1^H NMR (400 MHz, DMSO*-d_6_*) δ 8.23 (s, 1H), 7.93 (s, 1H), 7.80 (d, *J* = 8.2 Hz, 1H), 7.64 (d, *J* = 8.2 Hz, 1H),7.41 (s, 1H), 5.68 (s, 2H), 2.21 (s, 3H).

**PK6:** *N*-(5-(2-acetamidobenzo[*d*]thiazol-6-yl)-2-chloropyridin-3-yl)-2-phenylacetamide

To a stirred solution of PK6-5 (50 mg, 0.16 mmol) in pyridine (2 mL) at 0 °C was added 2-phenylacetyl chloride (30 mg, 0.19 mmol) dropwise. After carried out at room temperature overnight, the mixture was quenched with saturated aqueous NaHCO_3_ (1 mL). H_2_O (30 mL) was added and the resulting precipitate was filtered, washed with ethyl acetate to give a white solid (15 mg, 22 %). MP: 292-294 °C (decomposition); ^1^H NMR (400 MHz, DMSO*-d_6_*) δ 12.45 (s, 1H), 9.99 (s, 1H), 8.58 (s, 1H), 8.51 (s, 1H), 8.36 (s, 1H), 7.83 (d, *J* = 8.8 Hz, 1H), 7.74 (d, *J* = 8.8 Hz, 1H), 7.50-7.15 (m, 5H), 3.83 (s, 2H), 2.22 (s, 3H); ^13^C NMR (150 MHz, DMSO-*d_6_*) δ 170.6, 170.0, 159.5, 149.3, 143.6, 142.1, 135.9, 135.7, 133.0, 132.3, 132.0, 131.2, 129.7, 128.8, 127.1, 125.6, 121.4, 120.7, 43.0, 23.2; HRMS (ESI): calcd for C_22_H_17_ClN_4_O_2_S [M+H]^+^ 437.0834, found 437.0841.

1. **Synthesis of PK67**

**PK67-2:** *N*-(6-(5-amino-6-methylpyridin-3-yl)benzo[*d*]thiazol-2-yl)acetamide

A 25 mL flask was charged with PK6-3 (318 mg, 1.0 mmol), 5-bromo-2-methylpyridin-3-amine (187 mg, 1.0 mmol), K_2_CO_3_ (345 mg, 2.5 mmol) and Pd(PPh_3_)_4_ (90 mg, 0.08 mmol) followed by addition of dioxane/H_2_O (10 mL/0.5 mL). The equipment was evacuated and refilled with N_2_ three times. The reaction was carried out at 80 °C for 8 h. After cooled to room temperature, the solvent was concentrated and the residue was purified by silica gel column chromatography (dichloromethane/methanol = 50/1) to give the title compound as a white solid (150 mg, 50 %). ^1^H NMR (400 MHz, DMSO-*d_6_*) δ 8.13 (s, 1H), 8.00 (s, 1H), 7.73 (d, *J* = 8.4 Hz, 1H), 7.58 (d, *J* = 8.4 Hz, 1H), 7.20 (s, 1H), 5.14 (s, 2H), 2.30 (s, 3H), 2.18 (s, 3H).

**PK67**: *N*-(5-(2-acetamidobenzo[*d*]thiazol-6-yl)-2-methylpyridin-3-yl)-2-cyclohexylacetamide

A mixture of PK67-2 (30 mg, 0.10 mmol), 2-cyclohexylacetic acid (27 mg, 0.2 mmol), HATU (76 mg, 0.2 mmol) and TEA (30 mg, 0.3 mmol) in 2 mL DMF was stirred at room temperature overnight. The mixture was diluted with ethyl acetate ( 20 mL). The organic phase was washed with brine (10 mL*3). The organic phase was dried with Na_2_SO_4_ and the solvent was removed by vacuum. The residue was purified by column chromatography (dichloromethane/methanol = 100/2) to give a white solid (12 mg, 28 %). MP 287-288 °C (decomposition); ^1^H NMR (400 MHz, DMSO-*d_6_*) δ 12.41 (s, 1H), 9.51 (s, 1H), 8.62 (s, 1H), 8.31 (s, 1H), 8.12 (s, 1H), 7.82 (d, *J* = 8.4 Hz, 1H), 7.72 (d, *J* = 8.4 Hz, 1H), 2.45 (s, 3H), 2.29 (d, *J* = 6.8 Hz, 2H), 2.22 (s, 3H), 1.84-1.59 (m, 6H), 1.31-1.12 (m, 3H), 1.06-0.92 (m, 2H); ^13^C NMR (100 MHz, DMSO-*d_6_*) δ 171.4, 170.0, 159.1, 150.9, 148.9, 143.5, 133.7, 133.0, 132.7, 130.3, 125.4, 121.4, 120.2, 44.1, 35.3, 33.0, 26.3, 26.1, 23.3, 21.3; HRMS (ESI): calcd for C_23_H_26_N_4_O_2_S [M+H]^+^ 423.1849, found 423.1840.

1. **Synthesis of PK68**

**PK68-2**: cyclohexyl (4-nitrophenyl) carbonate

A mixture of PK68-1 (12 g, 118 mmol) and DMAP (1.2 g, 9.9 mmol) in THF (50 mL) was added dropwise 4-nitrophenyl carbonochloridate (20 g, 99 mmol) in THF (20 mL) at 0 °C. After the reaction was stirred for 4 h, the solvent was removed by vacuum. The residue was purified by silica gel column chromatography (petroleum ether/ethyl acetate = 50/1) to give a colorless oil (12.4 g, 47 %). ^1^H NMR(400 MHz, CDCl_3_) δ 8.27 (d, *J* = 8.0 Hz, 2H), 7.38 (d, *J* = 8.4 Hz, 2H), 4.83-4.69 (m, 1H), 2.09-1.92 (m, 2H), 1.87-1.73 (m, 2H), 1.68-1.59 (m, 2H), 1.49-1.26 (m, 4H).

**PK68-3**: cyclohexyl (5-bromo-2-methylpyridin-3-yl)carbamate

To a solution of 5-bromo-2-methylpyridin-3-amine (1.4 g, 7.6 mmol) in THF (20 mL) was slowly added NaHMDS (8 mL, 16 mmol) at 0 °C under N_2_. After 10 min, a solution of PK68-2 (2.4 g, 9.1 mmol) in THF (5 mL) was added slowly and stirred for 2 h. The reaction mixture was quenched by saturated aqueous NH_4_Cl (50 mL) and extracted with ethyl acetate (30 mL*3). The organic layers were combined and then dried over Na_2_SO_4_, filtered and concentrated. The residue was purified by silica gel column chromatography (petroleum ether/ethyl acetate = 10/1) to give the title compound as a yellow solid (2.3 g, 82 %). ^1^H NMR (400 MHz, CDCl_3_) δ 8.53 (br s, 1H), 8.27 (s, 1H), 6.40 (s, 1H), 4.83-4.70 (m, 1H), 2.49 (s, 3H), 2.00-1.91 (m, 2H), 1.80-1.71 (m, 2H), 1.63-1.53 (m, 2H), 1.50-1.36 (m, 4H).

**PK68**: cyclohexyl (5-(2-acetamidobenzo[d]thiazol-6-yl)-2-methylpyridin-3-yl)carbamate

A 25 mL flask was charged with PK68-3 (1.9 g, 6.3 mmol), PK6-3 (2.0 g, 6.3 mmol), K_2_CO_3_ (2.2 g, 15.7 mmol) and Pd(PPh_3_)_4_ (580 mg, 0.5 mmol) followed by addition of dioxane/H_2_O (44 mL/4 mL). The equipment was evacuated and refilled with N_2_ three times. The reaction was carried out at 80 °C for 8 h. After cooled to room temperature, the mixture was evaporated and the residue was purified by silica gel column chromatography (dichloromethane/methanol = 50/1) to give the title compound as a white solid (750 mg, 28 %). MP: 249-251 °C (decomposition); ^1^H NMR (400 MHz, DMSO-*d_6_*) δ 12.41 (s, 1H), 9.12 (s, 1H), 8.59 (s, 1H), 8.31 (s, 1H), 8.10 (s, 1H), 7.82 (d, *J* = 8.4 Hz, 1H), 7.72 (d, *J* = 8.4 Hz, 1H), 4.74-4.56 (m, 1H), 2.46 (s, 3H), 2.22 (s, 3H), 1.92-1.89 (m, 2H), 1.75-1.67 (m, 2H), 1.56-1.25 (m, 6H); ^13^C NMR (100 MHz, DMSO-*d_6_*) δ 169.5, 158.6, 153.9, 150.2, 148.4, 142.6, 133.3, 132.9, 132.5, 132.2, 129.0, 124.9, 120.9, 119.7, 72.8, 31.6, 24.9, 23.4, 22.8, 20.7; HRMS (ESI): calcd for C_22_H_24_N_4_O_3_S [M+H]^+^ 425.1642, found 425.1648.

1. **Synthesis of PK81**

**PK81-2**: cyclopentyl (4-nitrophenyl) carbonate

A mixture of PK81-1 (172 mg, 2 mmol) and DMAP (244 mg, 2 mmol) in THF (5 mL) was added dropwise 4-nitrophenyl carbonochloridate (404 mg, 2 mmol) in THF (2 mL) at 0 °C. After the reaction was stirred for 4 h, the solvent was removed by vacuum. The residue was purified by silica gel column chromatography (petroleum ether/ethyl acetate = 50/1) to give a colorless oil (200 mg, 40 %). ^1^H NMR(400 MHz, CDCl_3_) δ 8.27 (dd, *J* = 8.8, 1.6 Hz, 2H), 7.37 (dd, *J* = 8.8, 1.6 Hz, 2H), 5.26-5.16 (m, 1H), 2.00-1.73 (m, 6H), 1.71-1.61 (m, 2H).

**PK81**: cyclopentyl (5-(2-acetamidobenzo[d]thiazol-6-yl)-2-methylpyridin-3-yl)carbamate

A mixture of PK81-2 (200 mg, 0.8 mmol), Cs_2_CO_3_ (33 mg, 0.1 mmol) and PK67-2 (30 mg, 0.1 mmol) in DMF (1 mL) was stirred for 12 h at 120 °C. After cooling to room temperature, ethyl acetate (20 mL) was added and the resulting mixture was washed with brine (10 mL*3). The organic layer was dried over Na_2_SO_4_, filtered and concentrated. The residue was purified by silica gel column chromatography (dichloromethane/methanol = 50/1) to give a white solid (5 mg, 12 %). MP: 246-247 °C (decomposition); ^1^H NMR (400 MHz, DMSO*-d_6_*) δ 12.41 (s, 1H), 9.09 (s, 1H), 8.59 (s, 1H), 8.31 (s, 1H), 8.10 (s, 1H), 7.82 (d, *J* = 8.4 Hz, 1H), 7.72 (d, *J* = 8.4 Hz, 1H), 5.19-5.04 (m, 1H), 2.46 (s, 3H), 2.22 (s, 3H), 1.97-1.82 (m, 2H), 1.75-1.67 (m, 4H), 1.63-1.52 (m, 2H); ^13^C NMR (100 MHz, DMSO-*d_6_*) δ 170.0, 159.1, 154.7, 150.7, 148.9, 143.1, 133.8, 133.4, 133.0, 132.7, 129.5, 125.4, 121.4, 120.2, 77.6, 32.8, 23.8, 23.3, 21.2; HRMS (ESI): calcd for C_21_H_22_N_4_O_3_S [M+H]^+^ 411.1485, found 411.1486.

1. **Synthesis of PK-084**

**PK84-2**: *N*-(5-bromopyridin-2-yl)-4-methylbenzenesulfonamide

A mixture of PK-84-1 (20.0 g, 116 mmol) and TsCl (24.2 g, 127 mmol) in 80 mL pyridine was heated to 90 °C overnight. After cooled to room temperature, the solvent was removed by vacuum. 100 mL water was poured into the residue. The resulting precipitate was filtered. The cake was washed with water, dried over vacuum to give a white solid (35.5 g, 93 %). ^1^H NMR (400 MHz, DMSO*-d_6_*) δ 11.25 (br s, 1H), 8.27 (s, 1H), 7.89 (d, *J* = 8.0 Hz, 1H), 7.78 (d, *J* = 8.0 Hz, 2H), 7.36 (d, *J* = 8.0 Hz, 2H), 7.03 (d, *J* = 8.8 Hz, 1H), 2.35 (s, 3H).

**PK84-3**: (Z)-2-(5-bromo-2-(tosylimino)pyridin-1(2*H*)-yl)acetamide

A mixture of PK84-2 (35.5 g, 10 mmol), DIPEA (16.7 g, 129 mmol) and 2-iodoacetamide (23.9 g, 129 mmol) in 80 mL DMF was stirred at room temperature overnight. After that, 1 L water was added. The resulting precipitate was filtered. The cake was washed with water, dried over vacuum to give a grey solid (50.0 g, crude). ^1^H NMR (400 MHz, DMSO*-d_6_*) δ 8.38 (s, 1H), 7.87 (d, *J* = 9.6 Hz, 1H), 7.79 (s, 1H), 7.65 (d, *J* = 7.2 Hz, 2H), 7.41 (br s, 1H), 7.30-7.26 (m, 3H), 4.78 (s, 2H), 2.34 (s, 3H).

**PK84-4**: *N*-(6-bromoimidazo[1,2-*a*]pyridin-2-yl)-2,2,2-trifluoroacetamide

To a stirred solution of PK84-3 (18 g, 46 mmol) in 80 mL dichloromethane at room temperature was added TFAA (48.3 g, 230 mmol). The mixture was stirred at 60 °C overnight. After cooled to room temperature, the mixture was neutralized by sat. aq. NaHCO_3_ to pH = 7. The resulting precipitate was filtered. The cake was washed with water. The filtrate was extracted with dichloromethane (200 mL*3). The combined organic phases were dried over Na_2_SO_4_ and the solvent was removed by vacuum. The residue was recrystallized in petroleum ether to give a brown solid (15 g, crude). ^1^H NMR (400 MHz, DMSO-*d_6_*) δ 12.54 (s, 1H), 8.96 (s, 1H), 8.24 (s, 1H), 7.51 (d, *J* = 9.6 Hz, 1H), 7.41 (d, *J* = 8.8 Hz, 1H).

**PK84-5**: 6-bromoimidazo[1,2-*a*]pyridin-2-amine

A mixture of PK84-4 (15 g, crude, 46 mmol) in 1 N NaOH/EtOH (50 mL/40 mL) was stirred at 80 °C overnight. After cooled to room temperature, the aqueous phase was extracted with ethyl acetate (100 mL*3). The combined organic phases were dried over Na_2_SO_4_ and the solvent was removed by vacuum. The residue was recrystallized in petroleum ether to give a brown solid (8.3 g, 85%). ^1^H NMR (400 MHz, DMSO-*d_6_*) δ 8.60 (s, 1H), 7.32 -7.05 (m, 2H), 7.00 (s, 1H), 5.19 (s, 2H).

**PK84-6**: *N*-(6-bromoimidazo[1,2-a]pyridin-2-yl)acetamide

To a stirred solution of PK84-5 (552 mg, 2.6 mmol) and DMAP (350 mg, 2.9 mmol) in 20 mL dichloromethane at 0 °C was added Ac_2_O (290 mg, 2.9 mmol) dropwise. After carried out at room temperature overnight, the mixture was quenched with saturated aqueous NaHCO_3_ (40 mL). The resulting precipitate was filtered. The cake was washed with water and dried under vacuum to give a white solid (500 mg, 76 %). ^1^H NMR (400 MHz, DMSO-*d_6_*) δ 10.74 (s, 1H), 8.87 (s, 1H), 8.10 (s, 1H), 7.39 (d, *J* = 9.2 Hz, 1H), 7.30 (d, *J* = 9.2 Hz, 1H), 2.07 (s, 3H).

**PK84-7**: *N*-(6-(4,4,5,5-tetramethyl-1,3,2-dioxaborolan-2-yl)imidazo[1,2-a]pyridin-2-yl)acetamide

A 10 mL flask was charged with PK84-6 (510 mg, 2.0 mmol), bis(pinacolato)diboron (560 mg, 2.2 mmol), KOAc (590 mg, 6.0 mmol) and Pd(dppf)Cl_2_ (146 mg, 0.2 mmol) followed by addition of 5 mL DMSO. The equipment was evacuated and refilled with N_2_ three times. The reaction was carried out at 90 °C for 8 h. After cooled to room temperature, the mixture was filtered. The filtrate was diluted with ethyl acetate (40 mL). The organic phase was washed with brine (20 mL*3). The organic phase was dried with Na_2_SO_4_ and the solvent was removed by vacuum. The residue was recrystallized in petroleum ether to give a brown solid (500 mg, crude).

**PK84**: cyclohexyl (5-(2-acetamidoimidazo[1,2-a]pyridin-6-yl)-2-methylpyridin-3-yl)carbamate

A 25 mL flask was charged with PK84-7 (62 mg, 0.2 mmol), PK68-3 (60 mg, 0.2 mmol), K_2_CO_3_ (70 mg, 0.5 mmol) and Pd(PPh_3_)_4_ (23 mg, 0.02 mmol) followed by addition of dioxane/H_2_O (10 mL/0.5 mL). The equipment was evacuated and refilled with N_2_ three times. The reaction was carried out at 80 °C for 8 h. After cooled to room temperature, the solvent was evaporated and the residue was purified by silica gel column chromatography (dichloromethane/methanol = 50/1) to give the title compound as a white solid (12 mg, 15 %). MP: 268-269 °C (decomposition); ^1^H NMR (400 MHz, DMSO-*d_6_*) δ 10.73 (s, 1H), 9.15 (s, 1H), 8.96 (s, 1H), 8.55 (s, 1H), 8.13 (s, 1H), 8.07 (s, 1H), 7.52 (s, 2H), 4.70-4.57 (m, 1H), 2.45 (s, 3H), 2.08 (s, 3H), 1.95-1.90 (m, 2H), 1.80-1.66 (m, 2H), 1.57-1.50 (m, 1H), 1.49-1.26 (m, 5H); ^13^C NMR (100 MHz, DMSO-*d_6_*) δ 168.1, 154.4, 151.2, 142.8, 142.7, 140.7, 133.4, 130.9, 129.2, 124.5, 124.2, 121.8, 115.9, 101.5, 73.4, 32.1, 25.4, 23.9, 23.4, 21.2; HRMS (ESI): calcd for C_22_H_25_N_5_O_3_ [M+H]^+^ 408.2030, found 408.2032.

1. **Synthesis of PK86**

**PK86-2**: 2-bromo-5-methylpyridine 1-oxide

To a solution of PK86-1 (4.0 g, 23.2 mmol) in DCM (50 mL) was added *m*-CPBA (9.4 g, 46.4 mmol) slowly at 0 °C. After the reaction was stirred overnight, DCM (100 mL) was added. The solution was washed with saturated aqueous NaHCO_3_ (50 mL*3). The organic layer was dried over Na_2_SO_4_, filtered and concentrated. The residue was purified by silica gel column chromatography (dichloromethane/methanol = 50/1) to give the title compound as a brown solid (3.5 g, 85 %). ^1^H NMR (400 MHz, CDCl_3_) δ 8.24 (s, 1H), 7.52 (d, *J* = 8.4 Hz, 1H), 6.94 (d, *J* = 8.4 Hz, 1H), 2.28 (s, 3H).

**PK86-3**: 2-bromo-5-methyl-4-nitropyridine 1-oxide

To a mixture of H_2_SO_4_ (6 mL) and HNO_3_ (4.5 mL) was added slowly PK86-2 (3.5 g, 18.7 mmol) at 0 °C. The reaction was stirred for 2 h at 100 °C. After cooled to r.t, the reaction was poured into ice-water (20 mL). The resulting mixture was neutralized to pH 7 by saturated aqueous NaHCO_3_ and extracted with ethyl acetate (30 mL*3). The organic layers were combined, dried over Na_2_SO_4_, filtered and concentrated to give the title compound as a yellow solid (2.0 g, crude). ^1^H NMR (400 MHz, CDCl_3_) δ 8.40 (s, 1H), 8.29 (s, 1H), 2.58 (s, 3H).

**PK86-4**: 2-bromo-5-methylpyridin-4-amine

To a solution of PK86-3 (2.0 g, 8.6 mmol) in AcOH (20 mL) was added Fe (2.4 g, 43 mmol) slowly at 100 °C. The reaction mixture was stirred for 0.5 h and then filtered. The filtrate was concentrated by vacuum . The residue was diluted with ethyl acetate (30 mL) and adjusted pH to 7 by saturated aqueous NaHCO_3_, and extracted with ethyl acetate (30 mL*3). The organic layers were dried over Na_2_SO_4_, filtered and concentrated. The residue was purified by silica gel column chromatography (petroleum ether/ethyl acetate = 2/1) to give the title compound as a yellow solid (1.5 g, 93 %). ^1^H NMR (400 MHz, CDCl_3_) δ 7.85 (s, 1H), 6.69 (s, 1H), 4.16 (br s, 2H), 2.05 (s, 3H).

**PK86-5**: cyclohexyl (2-bromo-5-methylpyridin-4-yl)carbamate

To a solution of PK86-4 (374 mg, 2.0 mmol) in dry THF (20 mL) was slowly added NaHMDS (2 mL, 4 mmol) at 0 °C under N_2_. After 10 min, a solution of PK68-2 (1.1 g, 4.0 mmol) in dry THF (5 mL) was added slowly and stirred for 2 h. The reaction mixture was quenched by saturated aqueous NH_4_Cl (30mL) and extracted with ethyl acetate (30 mL*3). The organic layers were combined, dried over Na_2_SO_4_, filtered and concentrated. The residue was purified by silica gel column chromatography (petroleum ether/ethyl acetate = 7/1) to give the title compound as a yellow solid (170 mg, 21 %). ^1^H NMR (400 MHz, CDCl_3_) δ 8.26 (s, 1H), 8.03 (s, 1H), 6.57 (s, 1H), 4.79-4.74 (m, 1H), 2.17 (s, 3H), 2.00-1.90 (m, 2H), 1.80-1.70 (m, 2H), 1.60-1.55 (m, 2H), 1.53-1.33 (m, 4H).

**PK86**: cyclohexyl (2-(2-acetamidobenzo[d]thiazol-6-yl)-5-methylpyridin-4-yl)carbamate

A 25 mL flask was charged with PK86-5 (62 mg, 0.2 mmol), PK6-3 (63 mg, 0.2 mmol), K_2_CO_3_ (70 mg, 0.5 mmol) and Pd(PPh_3_)_4_ (23 mg, 0.02 mmol) followed by addition of dioxane/H_2_O (10 mL/0.5 mL). The equipment was evacuated and refilled with N_2_ three times. The reaction was carried out at 80 °C for 8 h. After cooled to room temperature, the mixture was concentrated and the residue was purified by silica gel column chromatography (dichloromethane/methanol = 33/1) to give the title compound as a white solid (8 mg, 9 %). MP: 227-228 °C; ^1^H NMR (400 MHz, DMSO-*d_6_*) δ 12.42 (s, 1H), 9.18 (s, 1H), 8.57 (s, 1H), 8.38 (s, 1H), 8.29 (s, 1H), 8.04 (d, *J* = 8.4 Hz, 1H), 7.80 (d, *J* = 8.4 Hz, 1H), 4.78-4.60 (m, 1H), 2.26 (s, 3H), 2.22 (s, 3H), 2.00-1.90 (m, 2H), 1.82 -1.71 (m, 2H), 1.62-1.16 (m, 6H); ^13^C NMR (100 MHz, DMSO-*d_6_*) δ 169.3, 158.7, 154.4, 153.2, 150.8, 149.0, 145.0, 134.3, 132.2, 124.5, 122.1, 120.5, 119.4, 111.3, 73.2, 31.5, 24.9, 23.4, 22.6, 14.6; HRMS (ESI): calcd for C_22_H_24_N_4_O_3_S [M+H]^+^ 425.1642, found 425.1642.

1. **Synthesis of PK93:**

**PK93-2**: 5-bromothiazolo[5,4-b]pyridin-2-amine

To a solution of KSCN (970 mg, 10 mmol) in AcOH (2 mL) was slowly added PK93-1 (348 mg, 2 mmol) at 0 °C. After 10 min, a solution of Br_2_ (420 mg, 2.6 mmol) in AcOH (1 mL) was added slowly and the final reaction mixture was stirred overnight at room temperature. The mixture was filtered and the filtrate was concentrated. The residue was adjusted to pH = 8 by saturated aqueous NaHCO_3_ and extracted with dichloromethane (30 mL*3). The organic layers were combined and dried over Na_2_SO_4_, filtered and concentrated. The residue was purified by silica gel column chromatography (dichloromethane/methanol = 100/1) to give the title compound as a white solid (190 mg, 41 %). ^1^H NMR (400 MHz, DMSO-*d_6_*) δ 7.95 (s, 2H), 7.56 (d, *J* = 8.4 Hz, 1H), 7.42 (d, *J* = 8.4 Hz, 1H).

**PK93-3**: N-(5-bromothiazolo[5,4-b]pyridin-2-yl)acetamide

To a stirred solution of PK93-2 (180 mg, 0.78 mmol) and DMAP (105 mg, 0.86 mmol) in 25 mL dichloromethane at 0 °C was added Ac_2_O (88 mg, 0.86 mmol) dropwise. The reaction mixture was run at room temperature overnight. The reaction mixture was quenched with saturated aqueous NaHCO_3_ (100 mL). The resulting precipitate was filtered. The cake was washed with water and dried to give a white solid (190 mg, 89 %). ^1^H NMR (400 MHz, DMSO-*d_6_*) δ 12.61 (s, 1H), 8.06 (d, *J* = 8.4 Hz, 1H), 7.67 (d, *J* = 8.4 Hz, 1H), 2.23 (s, 3H).

**PK93**: cyclohexyl (5-(2-acetamidothiazolo[5,4-b]pyridin-5-yl)-2-methylpyridin-3-yl)carbamate

A suspension mixture of PK68-3 (63 mg, 0.2 mmol), bis(pinacolato)diboron (59 mg, 0.2 mmol), Pd(dppf)Cl_2_ (15 mg, 0.02 mmol) and KOAc (49 mg, 0.5 mmol) in 5 mL of dioxane was stirred at 100 °C under N_2_ for 5 h. The reaction was filtered. The filtrate was added PK93-3 (27 mg, 0.1 mmol), Pd(PPh_3_)_4_ (13 mg, 0.01 mmol), K_2_CO_3_ (35 mg, 0.25 mmol) and H_2_O (1 mL). The reaction mixture was stirred at 80 °C overnight. The mixture was concentrated and the residue was purified by silica gel column chromatography (dichloromethane/methanol = 50/1) to give the title compound as a white solid (28 mg, 66 %). MP: 230-231 °C (decomposition); ^1^H NMR (400 MHz, DMSO*-d_6_*) δ 12.54 (s, 1H), 9.13 (s, 1H), 8.94 (s, 1H), 8.50 (s, 1H), 8.18 (d, *J* = 8.4 Hz, 1H), 8.09 (d, *J* = 8.4 Hz, 1H), 4.78-4.59 (m, 1H), 2.48 (s, 3H), 2.24 (s, 3H), 1.95-1.87 (m, 2H), 1.79-1.68 (m, 2H), 1.58-1.17 (m, 6H); ^13^C NMR (150 MHz, DMSO-*d_6_*) δ 172.5, 160.5, 158.1, 156.7, 154.6, 152.4, 145.3, 144.0, 135.7, 134.5, 131.3, 130.9, 121.3, 75.6, 34.3, 27.7, 26.1, 25.6, 23.7; HRMS (ESI): calcd for C_21_H_23_N_5_O_3_S [M+H]^+^ 426.1594, found 426.1594.
